# Technological progress in electronic health record system optimization: Systematic review of systematic literature reviews

**DOI:** 10.1016/j.ijmedinf.2021.104507

**Published:** 2021-08

**Authors:** Elsa Negro-Calduch, Natasha Azzopardi-Muscat, Ramesh S. Krishnamurthy, David Novillo-Ortiz

**Affiliations:** a World Health Organization Regional Office for Europe, Copenhagen, Denmark; b World Health Organization, Geneva, Switzerland

**Keywords:** AMSTAR, Assessment of Multiple Systematic Reviews, CA, Certificate Authority, CIM, Clinical Information Models, CIMI, Clinical Information Modelling Initiative, cTAKES, clinical Text Analysis and Knowledge Extraction System, DL, Deep Learning, EHR, Electronic Health Record, FHIR, Fast Healthcare Interoperability Resources, HiTEX, Health Information Text Extraction, HGD, Healthcare Data Gateway, HL7, Health Level 7, IE, Information Extraction, IHE, Integrating the Healthcare Enterprise, IoT, Internet of Things, ISO, International Organization for Standardization, LOINC, Logical Observation Identifiers Names and Codes, LSTM, long short-term memory model, MedLEE, Medical Extraction and Encoding, ML, machine learning, NLP, natural language processing, NLTK, Natural Language Toolkit, OBO, Open Biological and Biomedical Foundry adopted Symptom Ontology, pBFT, Practical Byzantine Fault Tolerance, PheWAS, Phenome-Wide Association Studies, PoW, Proof of Work, PRISMA, Preferred Reporting Items for Systematic Reviews and Meta-Analyses, PHR, personal health record, SNOMED-CT, Systemized Nomenclature for Medicine Clinical Terms, SVM, Support Vector Machines, UMLS, Unified Medical Language System, WHO, World Health Organization, WPM, words per minute, Medical informatics, Electronic health records, eHealth, Artificial intelligence, Blockchain, Phenotyping, Deep learning, Natural language processing

## Abstract

**Background:**

The recent, rapid development of digital technologies offers new possibilities for more efficient implementation of electronic health record (EHR) and personal health record (PHR) systems. A growing volume of healthcare data has been the hallmark of this digital transformation. The large healthcare datasets' complexity and their dynamic nature pose various challenges related to processing, analysis, storage, security, privacy, data exchange, and usability.

**Materials and Methods:**

We performed a systematic review of systematic reviews to assess technological progress in EHR and PHR systems. We searched MEDLINE, Cochrane, Web of Science, and Scopus for systematic literature reviews on technological advancements that support EHR and PHR systems published between January 1, 2010, and October 06, 2020.

**Results:**

The searches resulted in a total of 2,448 hits. Of these, we finally selected 23 systematic reviews. Most of the included papers dealt with information extraction tools and natural language processing technology (n = 10), followed by studies that assessed the use of blockchain technology in healthcare (n = 8). Other areas of digital technology research included EHR and PHR systems in austere settings (n = 1), de-identification methods (n = 1), visualization techniques (n = 1), communication tools within EHR and PHR systems (n = 1), and methodologies for defining Clinical Information Models that promoted EHRs and PHRs interoperability (n = 1).

**Conclusions:**

Technological advancements can improve the efficiency in the implementation of EHR and PHR systems in numerous ways. Natural language processing techniques, either rule-based, machine-learning, or deep learning-based, can extract information from clinical narratives and other unstructured data locked in EHRs and PHRs, allowing secondary research (i.e., phenotyping). Moreover, EHRs and PHRs are expected to be the primary beneficiaries of the blockchain technology implementation on Health Information Systems. Governance regulations, lack of trust, poor scalability, security, privacy, low performance, and high cost remain the most critical challenges for implementing these technologies.

## Highlights

• This review examined the literature on some digital advancements that may potentially impact and leverage in electronic health record systems.
• Existing digital transformation solutions have showed potential for optimizing electronic health records systems.
• Challenges for implementation remain such as lack of regulatory frameworks, trust, scalability, security, privacy, low performance, and cost.

## 1 Introduction

Recent developments in technology have impacted the digitalization of health data and facilitated the adoption of electronic health record (EHR) systems, which have become mandatory in some countries [1]. There are many definitions of Electronic Health Records (EHR). For the purpose of this paper we will use the broadly accepted ISO (International Organization for Standardization) definition [2]:

“An EHR is a data repository regarding the health and healthcare of a subject of care where all information is stored on electronic media”.

Furthermore, a personal health record (PHR) will be defined by ISO/TR 14292:2012(en) [3] as:

“A representation of information regarding, or relevant to, the health, including wellness, development and welfare of that individual, which may be stand-alone or may integrate health information from multiple sources, and for which the individual, or the representative to whom the individual delegated his or her rights, manages and controls the PHR content and grants permissions for access by, and/or sharing with, other parties.”

Growing volumes of healthcare data managed and stored electronically are inherent to the digital transformation [4]. The complexity and dynamic nature of large healthcare datasets pose challenges related to processing, storing, and analyzing such vast amounts of data. One of the main issues is that nearly 80 % of EHR data is unstructured (i.e., natural text language, diagnostic imaging) [5], making specialized data extraction tools necessary to derive meaningful information. Medical researchers cannot utilize the vast amount of health data locked in fragmented clinical databases to its full potential for personalized medicine and improved health outcomes [5]. Additionally, as more EHR data becomes accessible, more sophisticated methods are needed to safeguard data security and patient privacy (i.e., access-control policies, scrubbing, consent management). Blockchain technology is also gaining momentum in both industry and the public sector [[6], [7], [8], [9]].

With the rapidly changing digital landscape, it is important to develop an overview of the impact of digital solutions in individual health record systems, including both electronic and personal health records. The purpose of this systematic review of systematic reviews is to provide an overview and a summary of the challenges, opportunities, and implementation status of core technologies that may potentially impact and leverage EHRs and PHRs.

## 2 Materials and methods

### 2.1 Search strategy

Prior to conducting the review, we drafted a written protocol following the Preferred Reporting Items for Systematic Reviews and Meta-Analyses (PRISMA) [10] (Appendix A), including the review question, search strategy, methods, inclusion/exclusion criteria, risk of bias assessment, a synthesis plan, and a plan for investigating causes of heterogeneity.

Then, we performed a systematic literature search using MEDLINE (accessed by PubMed), Cochrane (Cochrane Database of Systematic Reviews, Cochrane Central Register of Controlled Trials, Cochrane Methodology Register, Database of Abstracts of Reviews of Effects, Health Technology Assessment, Evidence-Based Practice Center program, National Health Service Economic Evaluation Database), Web of Science (including SciELO Citation Index, Current Contents Connect, KCI-Korean Journal Database, Russian Science Citation Index) and Scopus. A manual search of references supplemented the database search. Instead of using a combination of EHRs and digital technologies keywords, we chose a more sensitive search strategy by using terms only related to EHRs and PHRs. Although this choice required more studies to be screened (n = 2,448), we purposefully intended to minimize the risk of missing any potentially eligible studies. There were no significant deviations from the protocol. The search strategies for each database are available in Appendix B. We identified keywords through a preliminary search (Textbox 1).

Textbox 1. Search terms

**Table:** 

**Search terms related to health technologies**: health informatics OR mobile health OR mobile phone applications OR smartphone OR apps OR telemedicine OR interoperability OR Internet OR digital health literacy OR social media (Facebook, Twitter, YouTube, Instagram, Flickr, Google, LinkedIn, blog, wiki) OR big data OR open data OR personalized medicine OR data mining OR wearable OR smart health OR internet of things OR Wireless Technology OR cloud OR Bluetooth OR eHealth OR digital health OR information and communication technology OR SMS OR blockchain OR data science OR artificial intelligence OR machine learning OR deep learning **Search terms related to electronic health records**: individual health record OR electronic medical records OR electronic personal health record OR digital record OR health record OR personal health record OR medical record system OR electronic health record.

### 2.2 Eligibility criteria and study selection

We searched for systematic literature reviews where the intervention was any digital solution, and the outcome was the intervention’s impact on personal and electronic health records. Articles published between January 2010 and October 2020 in English, French, Spanish, Italian, and Portuguese were eligible. Exclusion criteria were (1) studies not relevant to the purpose of the review, (2) studies about usability or user acceptance, (3) studies about the impact of EHRs or personal health records on specific health outcomes, (4) studies with a focus on privacy and cyberthreats, (5) studies on legislation related to EHRs, (6) non-systematic reviews, (7) studies written in another language, (8) studies published before 2010, (9) non-human studies, or (10) studies for which full-text was not available. We imported references into Endnote X7.8 (Thomson Reuters, Toronto, ON, Canada) and removed duplicates. The selection process took place in two steps. First, we screened titles and abstracts applying the criteria above. Secondly, we reviewed the full text of articles retrieved in the first step. The screening and full-text review were carried out independently by two reviewers. Studies were selected once a consensus was reached.

### 2.3 Data collection process

Data from the full-text selected papers were exported into MS Excel by a single reviewer and verified by a second reviewer. We extracted the following data: journal, publication year, databases searched, study period, setting/scenario, purpose, intervention type, number of studies, study design, main results, opportunities, and implementation challenges.

### 2.4 Quality assessment

We contemplated using the assessment of multiple systematic reviews tool (AMSTAR2) [11] for assessing methodological quality. However, since descriptive studies are not the primary target of AMSTAR2, we found the applicability of some checklist items unclear. Moreover, consensus on quality assessment tools for descriptive studies is still lacking [[12], [13], [14]]. Therefore, we deviated from the original AMSTAR2 checklist and adapted the list of questions, which better fit the methodological focus of the selected systematic reviews. The tailored checklist for assessing the quality of the selected studies is available in Table 1.

**Table 1:** Quality assessment criteria.

Question number	Issue
Q1	Did the review clearly show the purpose of the research?
Q2	Did the review adequately describe the literature review, background, or context?
Q3	Did the review authors use a comprehensive literature search strategy?
Q4	Did the review authors perform study selection in duplicate?
Q5	Did the review authors perform data extraction in duplicate?
Q6	Did the review authors provide a list of excluded studies and justify the exclusions?
Q7	Did the review authors describe the included studies in adequate detail?
Q8	Was the scientific quality of the individual studies assessed?
Q9	Did the review authors provide a satisfactory explanation for, and discussion of, any heterogeneity observed in the results?
Q10	Did the review authors report any potential conflict of interest?
Q11	Did the review authors report on sources of funding?

## 3 Results

**Fig. 1:**
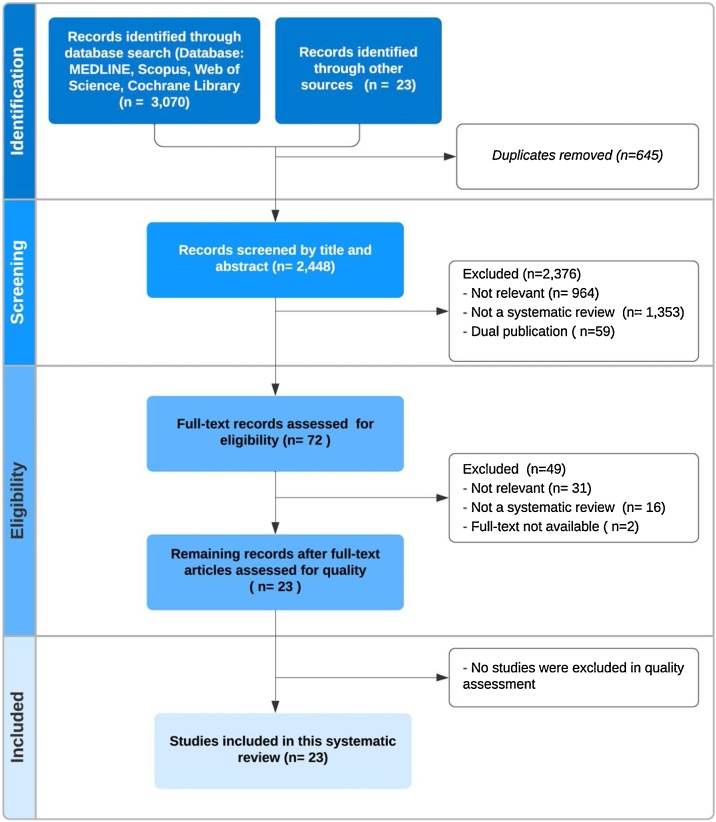
PRISMA flow chart of the systematic review of systematic reviews on the impact of technological advancements on electronic health record systems.

A flow chart of the literature search and study selection results is shown in Fig. 1.

A total of 3,093 articles were retrieved. After removing duplicates, we screened 2,448 records by title and abstract. Of these, 964 (39.4 %) articles were not relevant to the research question. We identified and removed another 59 overlapping publications. Seventy-two full-text articles were reviewed, and 23 systematic reviews were selected for the study [[15], [16], [17], [18], [19], [20], [21], [22], [23], [24], [25], [26], [27], [28], [29], [30], [31], [32], [33], [34], [35], [36], [37]]. An overview of all selected studies is presented in Table 2, with the first author, year of publication, journal, and research area. The list of 49 studies that were excluded after full-text review with reasons for exclusion can be found in Appendix C.

**Table 2:** List of selected studies organized by Area.

Author, year	Journal	Area
Dainton et al., 2017 [16]	J. Med. Internet Res.	EMRs for austere settings
West et al., 2015 [32]	J. Am. Med. Inform. Assoc.	EHR visualization tools
Moreno-Conde et al., 2015 [26]	J. Am. Med. Inform. Assoc.	Clinical Information Models (CIMs)
Meystre et al., 2010 [24]	BMC Med. Res. Methodol.	De-identification
Walsh et al., 2013 [30]	J. Med. Internet Res.	Provider-to provider electronic communication tools
Dubovitskaya et al., 2020 [17]	Oncology	Blockchain Technology
Hasselgren et al., 2020 [18]	Int. J. Med. Inform.	Blockchain Technology
Mayer et al., 2020 [23]	Health Informatics J.	Blockchain Technology
O'Donoghue et al., 2019 [27]	J. Med. Internet Res.	Blockchain Technology
Chukwu et al., 2020 [37]	IEEE Access	Blockchain Technology
Mazlan et al., 2020 [36]	IEEE Access	Blockchain Technology
Hussien et al., 2019 [35]	J. Med Syst.	Blockchain Technology
Vazirani et al., 2019 [29]	J. Med. Internet Res.	Blockchain Technology
Mishra et al., 2014 [25]	J. Biomed. Inform.	Information Extraction/ Natural Language Processing (NLP)
Juhn et al., 2020 [19]	J. Allergy Clin. Immunol.	Information Extraction/ Natural Language Processing (NLP)
Koleck et al., 2019 [20]	J. Am. Med. Inform. Assoc.	Information Extraction/ Natural Language Processing (NLP)
Kreimeyer et al., 2017 [21]	J. Biomed. Inform.	Information Extraction/ Natural Language Processing (NLP)
Wang et al., 2020 [31]	J. Biomed. Inform.	Information Extraction/ Natural Language Processing (NLP)
Kumah-Crystal et al., 2018 [22]	Appl. Clin. Inform.	Information Extraction/ NPL/ Speech recognition (SR)
Blackley et al., 2019 [15]	J. Am. Med. Inform. Assoc.	Information Extraction/ NPL/ Speech recognition (SR)
Shivade et al., 2014 [28]	J. Am. Med. Inform. Assoc.	Information Extraction/ NLP/ Phenotyping
Xu et al., 2015 [34]	J. Am. Med. Inform. Assoc.	Information Extraction/ NLP/ Phenotyping
Xiao et al., 2018 [33]	J. Am. Med. Inform. Assoc.	Information Extraction/ NLP/ Deep learning

Searching for multiple systematic reviews on the same topic definitively resulted in some overlap. Specifically, 8 out of the 23 studies included, which represented nearly 35 % of all systematic literature reviews, dealt with the impact and uses of blockchain technology in EHR/PHR. When checking for overlap we found that of the 332 studies included in these 8 reviews, there were 70 duplicate citations and 262 unique studies. On further analysis we still considered all 23 studies qualified for inclusion.

### 3.1 Quality of included studies

The proposed quality criteria scores were assessed for each selected article. Although none of the items fully satisfied the eleven criteria for quality assessment, all articles clearly presented their research purpose, used a comprehensive search strategy, described the background and included papers in detail, and discussed any heterogeneity found. No reviews provided a list of excluded studies during full-text screening, but two provided the full list of studies included at each step of the screening process. Twelve reviews did not use any method for quality assessment. Most reviews (20/23) reported any potential conflicts of interest and funding sources (20/23). Appendix D shows the results of the quality assessment of the 23 systematic reviews.

### 3.2 Study characteristics

All included papers were published between 2013 and 2020 in 11 journals. Considering the format and content of each review, we classified each into seven general classes. Most studies (n = 10) assessed information extraction (IE) and natural language processing technology (NLP) followed by studies that evaluated the use of blockchain technology in healthcare (n = 8). Other areas included digital solutions for EHR systems in austere settings of low-income countries (n = 1), de-identification methods (n = 1), visualization techniques for EHR data (n = 1), communication tools within EHR systems between healthcare providers (n = 1), and the processes and methodologies for defining Clinical Information Models (CIM) that promote EHR interoperability (n = 1). None of the systematic reviews performed a meta-analysis. The analysis of results is shown in Table 3, where the potential impact of the technical solutions, opportunities, and the outstanding issues to be addressed for each of these tools is available. In addition, a descriptive summary of the 23 systematic reviews included is in Appendix E.

**Table 3:** Opportunities, challenges, and technical solutions of EHR technological advancements identified in the review.

Digital tool	Opportunities	Challenges	Technical solutions
Blockchain technology	•Improved interoperability and data exchange amongst providers and patient-providers. •Improved data access •Consensus and immutability. •Potential improved operating efficiency. •Improved security of medical data stored in EHRs. •Improved health outcomes.	•Poor scalability. •Low general performance. •High cost. •Maintaining data privacy. •Security vulnerabilities. •Block size. •High volume of data. •Number of nodes. •Protocol challenges. •Regulatory frameworks. •Lack of education and trust. •Agreement and consensus between network participants is needed	•Decentralization of medical database. •Cryptographic techniques. •Blockchain authentication and authorization. •Storage optimization (mini blockchain; VerSum; Reference pointer FHIRChain). •Blockchain modeling (FHIRChain; HealthChain; DeepLinQ; OmniPHR). •Read mechanism (Short-term data sharing, Catching system). •Write mechanism (Smart contract; Cohort algorithm; Tokenization; Sharding; Practical Byzantine. Fault Tolerant consensus protocol; TrustChain). •Bi-directional (Lightning network).
Advanced visualization	•Knowledge discovery. •Better communication of information about EHR data.	•Large EHR datasets. •Temporal complexity, diversity, and evolving nature of EHR data. •Outdated visualization techniques. •Low data quality and completeness.	•LifeLines. •KNAVE-II/VISITORS. •Methods developed by other disciplines (i.e., computer science, engineering, and genetics) should be explored for their use with EHR data.
EHR systems in austere settings	•EHR systems are needed in austere settings where transport and storage of paper-based records are not feasible. •Improved data integrity, quality, and completeness. •Improved diagnosis and clinical management of patients. •Consistent standard of practice on medical service trips. •Better epidemiological analysis.	•The setting only allows connectivity through expensive satellite connections, opportunistic internet connections, or local networks. •There are multiple EHR systems for austere settings, and most are still at the development stage. •Limited or no interoperability.	•OpenMRS has potential to integrate MST medical records with local EHR systems. •Competing smaller EHR systems should consider further development for improved interoperability (i.e., iChart. SmartList To Go, Project Buendia, TEBOW, OpenMRS software, QuickChart EMR, NotesFirst).
Clinical Information Models (CIM)	•CIMs allow for semantic and structural interoperability of data between different EHR systems.	•Different technologies and standards (e.g., EN ISO 13,606 and openEHR, using archetypes, or HL7 v3, using templates) are being used. •Immaturity of current modelling support tools.	•Share CIMs openly. •Harmonize work amongst groups developing CIMs. •A common standard and a unified good practice methodology for CIMs needs to be developed.
De-identification tools	•Data privacy preservation.	•The negative impact of de-identification on subsequent automated information extraction.	•Machine learning-based methods based on: oConditional Random Fields, oDecision Trees, oMaximum Entropy models, or oSupport Vector Machines.
Natural Language Processing/free-text processing	•Enable secondary use of EHRs for phenotyping, clinical, translational research and implementation of personalized medicine. •Leverage unstructured data locked in EHRs •Support clinical management for better outcomes.	•Poor data quality, errors and biases. •Privacy issues. •Predominance of rule-based over machine learning-based NLP. •Difficult interpretability of machine-learning methods. •Algorithmic bias. •Lack of interoperabie standards. •Poor generalizability. •Developing NLP talent is difficult due to the limited availability and exposure of NLP experts to EHR data.	•Develop deep-learning based NLP for EHR data mining. •Share NLP algorithms publicly on platforms such as GitHub to avoid duplication and improve development. •Further development of ontologies such as the Open Biological and Biomedical Foundry.
NLP/ Speech Recognition (SR) technology	•Improved usability of EHR systems. •Improved productivity. •Better quality of clinical documentation (copy/paste behaviour is reduced). •Reduced workload for clinicians	•Low report accuracy and more errors in SR based documentation. •Significant upfront costs derived from SR introduction.	•Use of deep learning. •Potential for EHR-integrated, SR virtual assistants powered by AI. Consumer voice tools technology (i.e., Siri, Alexa) could be applied to EHR systems.
Deep learning	•Disease detection/classification. •Prediction of clinical events. •Phenotyping. •Data augmentation. •EHR data privacy/de-identification.	•Temporality and irregularity of EHR data. •Multimodal EHR learning is challenging due to the heterogeneity of the data. •Identifying effective ways to label EHR records is a major obstacle. •Lack of interpretability and transparency.	•Gated architecture for extracting temporal data. •Dynamic time warping, and a subspace decomposition of the Long short-term memory model (LSTM) to solve challenges associated with time irregularity. •Multitask learning approaches. •Transfer learning to new datasets for the same tasks. •Attention-mechanism-based learning, knowledge injection and knowledge distillation.

### 3.3 Digital solutions for EHR systems in austere settings

One systematic review assessed technological EHR solutions specifically designed for mobile medical missions working in austere conditions [16]. Their comprehensive search only yielded two publications, each describing a system (iChart, SmartList To Go) [38,39]. Another thirteen EHR systems were found through internet searches. Three (Project Buendia, TEBOW, and a University of Central Florida's internally developed EMR) were based on modified versions of OpenMRS software, whereas another three were smartphone apps (QuickChart EMR, iChart, NotesFirst). The availability of numerous independent EMR systems may further fragment medical care in low resource settings. An important caveat of these systems related to limited internet access. Connectivity can only be guaranteed through expensive satellite connections, opportunistic internet connections or local networks, which sometimes exceed logistic capabilities existing in those settings. Most EHR systems for austere settings are still at the pilot phase, and further development is needed. Specifically, interoperability and data sharing with larger systems should be considered a priority before widespread implementation.

### 3.4 Clinical Information Models

Clinical Information Models (CIMs) are technical specifications that define how clinical information is managed inside an EHR system, determining how interoperable EHR systems are. One review [26] identified and compared processes and methodologies for defining CIMs that promoted EHR interoperability. Only 52.8 % of the included studies described the CIM process, and only 11.1 % provided a detailed description of the terminology binding process. The authors recommended sharing CIMs openly.

Despite using different technologies and standards (e.g., EN ISO 13606 and openEHR), all reviewed papers used a similar methodological approach to create CIMs. Thus, the review showed the possibility of developing a common standard and a unified best practice methodology for CIMs supporting EHR interoperability. The review did not find a unified standard CIM process in the literature. At the time, the Clinical Information Modelling Initiative (CIMI) was in a conceptual stage.

### 3.5 De-identification methods

De-identification scrubs patient identifiers while safeguarding relevant health data. Eighteen methods for automatically de-identifying narrative text in EHR were reviewed by Meystre, et al. [24]. The methods used were Conditional Random Fields, Decision Trees, Maximum Entropy models, or Support Vector Machines (SVM), combined with dictionaries and sometimes regular expressions. Most identified methods used pattern matching, rules, and dictionaries instead of machine learning-based methods. However, machine learning (ML) methods, and specially deep-learning, have shown a better performance [33]. No studies assessed the impact of de-identification on subsequent automated information extraction despite that technologies such as NLP may be less successful when processing de-identified reports compared to fully identified reports.

### 3.6 Communication tools within EHR systems between healthcare providers

Walsh et al. reviewed electronic communication tools between healthcare providers, both within and external to EHR systems [30]. The authors assessed to what extent EHRs might impede effective communication; however, data on unintended consequences of provider-to-provider electronic communication were limited. The most reported tools were electronic referrals to specialty providers, electronic prescribing, and messaging. Most studies reported on measures of usability and adoption. Disadvantages of EHR communication related to the decrease in face-to-face interactions and knowledge sharing, which become critical in emergencies or with complex patients. Ideally, EHR systems should ensure implicit and real-time provider-to-provider communication.

### 3.7 Visualization techniques for EHR data

One review investigated the use of innovative visualization techniques for complex, longitudinal big data in EHR systems [32]. The most common visualization techniques reported in the literature were LifeLines and KNAVE-II/VISITORS. Although other disciplines, such as engineering, and genetics, have already developed advanced visualization tools for displaying complex big data, healthcare is lagging in adopting these techniques.

### 3.8 Blockchain technology

Eight reviews covered blockchain-based EHR systems. Blockchain is a decentralized solution for data storage designed for multiple users. A peer-to-peer network of nodes processes transactions, and a copy of the entire ledger is shared across all participants, who hold the whole database. All reviews assessed publications, addressing blockchain architectures, storage schemes, ontologies, privacy/security, performance, cost, data sharing, access control, audit, integrity, distributed computing, digital health standards, and data aggregation [17,18,23,27,29,[35], [36], [37]]. The majority of publications were still conceptual and used simulation software for their study evaluation.

Ethereum platform, hyperledger fabric, Proof of Work (PoW), exonum, and Practical Byzantine Fault Tolerance (pBFT) were the most commonly used platforms [18,27,37]. However, researchers highlighted the need for developing a new platform designed explicitly for EHR requirements [18] and standardization of EHR semantics, ontologies, standards, and technical approaches [23,27,29].

One review classified blockchain architectures implemented in healthcare according to the type of Certificate Authority (CA) used [37]. CAs provide identity on the network, signing requests for all entities and components. The three types of architectures identified were: individually managed, trusted-CA managed, or multi-CA managed. Individually managed CAs (patient or provider) lead to more complicated data integrity and security. Poor scalability, low general performance, and high associated costs were identified as critical challenges for blockchain implementation in healthcare [37].

Internet of Things (IoT) devices cause security vulnerabilities [37]. The decentralized feature of blockchain allows for better data sharing and medical data management while ensuring data integrity. Blockchain authenticates and authorizes users, preventing system threats to specific security attacks but cannot alone guarantee data privacy and security. Blockchain-based systems rely on cryptographic methods to maintain security [36]. The development of quantum computing might challenge those cryptographic techniques as quantum-resistant cryptography has not been developed yet. One review discussed whether health providers should wait and invest in a post-quantum blockchain [27].

Blockchain can also solve issues related to interoperability and medical data exchange amongst different healthcare providers, specifically in combination with open standard Fast Healthcare Interoperability Resources (FHIR), a standard for exchanging EHRs [36,37]. Other solutions include a blockchain-based app called Healthcare Data Gateway (HGD), which could potentially improve data sharing while guaranteeing patients’ privacy [36]. OmniPHR blockchain-based architecture also integrates personal health records (PHR), improving data exchange between patients and healthcare providers [36].

Blockchain for data sharing in oncology was reviewed, however, there have been no implementations in real-world settings due to obstacles related to regulatory frameworks, required consortium membership, poor interoperability, and data safety and privacy [6].

Finally, blockchain scalability challenges relate to the large amount of health data stored in EHRs [36]. In practice, EHR big data means large block sizes, many transactions, and an increased number of nodes representing each entity connected to the network (i.e., patients). Protocols must be tailored to satisfy the latency and throughput requirements to achieve an efficient performance [[35], [36], [37]]. Protecting the security and privacy of data is still associated with an unsatisfactory performance [6,27].

A balance must be struck between data protection and patients’ and providers' abilities to access and interact regularly with data [37]. One proposal is that only essential data for specific nodes are stored in-chain [36]. Implementers should also carefully assess scalability to prevent related issues [27]. Other solutions for storage optimization and for redesigning blockchain are summarized in Table 3.

### 3.9 Tools for information extraction from EHR

EHRs are comprised of data in a structured format (i.e., laboratory test results) and unstructured free-text narratives (i.e., notes or images), which constitute 80 % of currently available health data [5,19]. Besides, increasing amounts of unstructured data are becoming available through online patient portals, as communication between clinicians and patients is largely done in free text [19]. Due to the challenges related to its processing and extraction, unstructured data available in EHRs is seriously underutilized despite the high value for clinical and translational research, to define phenotype, characterize or classify disease, or even enable virtual clinical cohorts [19,40]. Ten of the selected studies reviewed tools for Information Extraction (IE) from EHR systems. Two of them specifically addressed approaches for identifying patient phenotype cohorts from EHRs [28,34]. Five [[19], [20], [21],^25^,^31^] focused on NLP. Two reviews assessed speech recognition, also a part of NLP [15,22]. Finally, Xiao et al. [33] reviewed deep learning (DL) models using EHR data [41].

IE is an interdisciplinary field of medicine and computer science, part of NLP. NLP is a subset of Artificial Intelligence (AI), which deals with how computers identify and translate written or spoken human language into machine-readable formats.

Methods for Information Extraction (IE) can be divided into rule-based and machine learning approaches [31]. Rule-based methods consist of handcrafted expressions that define a pattern of properties that need to be fulfilled. Rules are developed by manual knowledge engineering, by leveraging knowledge bases (i.e., UMLS, PheWAS), or through a combination of both. Manual knowledge engineering is accurate but requires collaboration with clinical experts and is time-consuming. Rule-based approaches, like those developed by large vendors (i.e., IBM, Microsoft), dominate IE because they are easier to use and yield good results on limited datasets [20,25,28,31].

However, ML approaches perform better and are deemed more appropriate and less time-consuming when handling big data [20,28,31]. The ML methods most commonly employed were Support Vector Machine (SVM) followed by Conditional random field (CRF) and are mostly used for data prediction [31].

Overall, the most popular IE tools were Apache cTAKES, MetaMap, Medical Language Extraction, Encoding system (MedLEE), TextHunter, and Multi-threaded Clinical Vocabulary Server, and the v3NLP Framework [19,25,31]. Major disease areas for IE use were cancer, followed by cardiovascular disease [20,31].

One major challenge of IE systems is their poor portability, primarily due to the multidimensionality of medical language, the lack of standardization, and the heterogenicity across EHR systems [20,31]. IE tasks are usually defined without standard information models or value sets [31]. Moreover, poor data quality, biases, and errors hamper the ability of NLP to recognize and process data [[19], [20], [21]]. Interoperable ontologies such as SNOMED-CT, or the Open Biological and Biomedical Foundry adopted Symptom Ontology (OBO) support the application of NLP and need to be widely adopted [20,31]. Open-source EHR-related NLP systems and making expert-developed NLP algorithms publicly available on platforms such as GitHub could avoid duplication and speed up NLP development [20].

### 3.10 Speech recognition

Speech is much faster than writing for data entry. A person speaks an average of 110–150 words per minute (WPM) compared to 40 WPM typing speed [22]. Furthermore, studies show that humans are more comfortable with handheld devices, which are portable and easier to handle [15]. Speech recognition (SR) technology could potentially improve workflow inefficiencies and assist clinical documentation through dictation. As an example, Vocera has been used to initiate phone calls, review messages, and authenticate logins through voice commands [15,22]. However, five studies reported a decrease in documentation time, nine an increase, and four found no impact [15]. Productivity typically improved, while report accuracy was lower after SR adoption, with more errors in SR-based documentation [15,22]. One study reported that 23 % of SR reports contained errors, compared to only 4 % created with conventional dictation or speech transcription [22]. Background noise, accents, and interruptions challenged the accuracy and utility of SR. On the other hand, SR technology reduced copy/paste behavior from 92.73 % to 49.71 %, leading to higher quality reports. With recent developments in AI, enormous potential exists for EHR-integrated SR virtual assistants for data retrieval, command execution, and chart navigation. Long short-term memory (LSTM), an artificial recurrent neural architecture used in deep learning, will bring significant improvements in this area [33].

### 3.11 Phenotyping

In the last years, there has been a rise in cohort identification studies that use EHR data, and information extraction for phenotyping has accounted for a large portion of the reviewed studies [31]. Two reviews assessed automated phenotyping techniques [28,34]. Xu et al. identified twenty-four EHR-driven phenotype algorithm authoring tools. These tools provide an interface to clinical researchers to define the algorithm criteria for determining patient cohorts without them needing to use a programming language [34]. Many of these tools did not support complex logic specifications nor external analytic software, and only 44 % of them could process unstructured data [28,34]. Rule-based systems were also dominant in phenotyping studies [28]. Overall, the reviews found that phenotyping techniques were still inadequate for the task. A significant challenge was the lack of portability between institutions. Standardization terminology systems such as RxNorm, SNOMED-CT, and LOINC, are not comprehensive for complex phenotype algorithms [28]. A standard mechanism for phenotype algorithm representation still needs to be developed [28,34].

### 3.12 Deep learning

One review summarized all DL studies using EHR data for disease detection/classification, prediction of clinical events, phenotyping, data augmentation, and EHR data privacy/de-identification [33]. DL is a subset of machine learning that applies artificial neural networks to learn a procedure. DL approaches require less manual engineering and minimal pre-processing. Commonly used DL architectures include feedforward neural networks, recurrent neural, restricted Boltzmann machines, generative adversarial networks, convolutional neural networks, word2vec, and denoising autoencoders. Outstanding challenges relate to the temporality and heterogenicity of EHR data and labelling EHR records. Long short-term memory models (LSTM) or gated recurrent units are the preferred choices for extracting long-term temporal data. Moreover, there are important issues concerning the transparency and interpretability of DL models. Users still need to understand the mechanisms by which models operate.

## 4 Discussion

In this study, we have provided an overview of the technological advancements developed in the last decade to support EHR systems’ optimization. Most selected papers (18 out of 23) were related to EHRs data-extraction tools and blockchain technology. Many methods for extracting EHR data have been assessed and published during the last decade. However, deep learning has become the preferred approach because it yields better performance in processing and modelling vast amounts of data while requiring less manual engineering [33].

Beyond the obvious advantages of digitizing health data, the adoption of EHRs has contributed to physician burnout [42,43]. Most physicians have reported feeling pressure related to filling out EHRs documents and spending excessive time on EHRs at home [43]. NLP tools, including speech recognition virtual assistants, have the potential to save clinicians valuable time [15,22].

Blockchain technology could resolve many interoperability issues, empowering patients with greater control of their data and privacy. However, blockchains are designed for consortia, not for a single organization. Agreements, trust, and consensus between network participants are needed. Furthermore, blockchain security vulnerabilities and performance issues still need to be addressed [[35], [36], [37]].

By restricting our review to EHR-related terms, we may have excluded meaningful studies on digital advancements that may also impact EHR systems. Furthermore, some papers might have been missed due to the lack of access to databases containing more technical research such as ACM and IEEE Explore. In addition, since we focused on published literature, we excluded many unpublished technological advancements and real-world implementations. For example, Estonia became the first country to use blockchain to secure its nationwide EHR system [44,45]. The World Health Organization (WHO) and the Estonian government are currently developing a blockchain-based COVID-19 vaccination certificate platform to be used globally [46,47].

Most of these tools are in very early stages and will benefit from maturity. Widely accepted health data standards are essential to ensure seamless data sharing, better coordination, and improved interoperability. The outstanding issues identified in this review (see Table 3) must be addressed before witnessing the full impact of these technological advancements on EHRs and knowing to which extend these tools will meet our expectations.

## Authors’ contributions

DNO, ENC and NAM conceptualized the study and designed the review. DNO and ENC developed the search strategies and performed screening, selection, and data extraction. DNO and ENC checked the accuracy of extracted data. ENC wrote the first draft of the review paper. DNO, ENC, NAM, and RSK contributed to the final draft of this manuscript. All authors read and approved the final manuscript.

**“What was already known on the topic”**

• Recent developments in technology have impacted the digitalization of health data, facilitating the adoption of electronic health record (EHR) systems.
• A growing volume of healthcare data managed and stored electronically remains underutilized for clinical and translational research.
• As more EHR data becomes accessible, several digital tools are gaining momentum in both the industry and the public sector.

**“What this study added to our knowledge.”**

• It provides a comprehensive overview of core technologies that may potentially impact and leverage electronic health record systems, with associated opportunities, challenges, and solutions.
• There is a growing trend in natural language processing, data mining extraction applications, and promising use cases for blockchain technology in healthcare; however, challenges such as immaturity, data privacy, poor scalability, and poor interoperability still need to be addressed.

## Disclaimer

The authors alone are responsible for the views expressed in this publication and those views do not necessarily represent the views, decisions or policies of the World Health Organization.

## Declaration of Competing Interest

The authors report no declarations of interest.
