# Supplementary material for: Technological progress in electronic health record system optimization: Systematic review of systematic literature reviews
Source: Int J Med Inform. 2021 Aug;152:104507. doi: 10.1016/j.ijmedinf.2021.104507 (PMC8223493; doi:10.1016/j.ijmedinf.2021.104507)

**Protocol for the Review of Systematic Reviews on the Impact of Digital Solutions in Individual Health Record Systems**

Prepared by Elsa Negro Calduch, WHO consultant

Table of Contents

[Background 2](#_Toc53268394)

[Methodology 2](#_Toc53268395)

[OBJECTIVES 2](file://Users/piki/Library/Containers/com.microsoft.Word/Data/Desktop/Deliverable%202.%20Review/Protocol.Review.Digitalsol.v.02.docx#_Toc53268396)

[SEARCH STRATEGY 3](file://Users/piki/Library/Containers/com.microsoft.Word/Data/Desktop/Deliverable%202.%20Review/Protocol.Review.Digitalsol.v.02.docx#_Toc53268398)

[PROTOCOL FOR DATA EXTRACTION 7](#_Toc53268399)

[Data Extraction and Quality Assessment 7](#_Toc53268400)

[Data Management and Storage 8](#_Toc53268401)

[References 9](#_Toc53268402)

[Annex 1: Search Strategy 10](#_Toc53268403)

- Current Version: 11/10/2020 -

# Background

A health technology is defined by the WHO as “the application of organized knowledge and skills in the form of medicines, medical devices, vaccines, procedures and systems developed to solve a health problem and improve quality of life”^1^. Digital healthcare solutions like mHealth, artificial intelligence (AI), and robotics have brought with them a great potential to further develop healthcare services. The impact of digitalization of health services has been profound and is expected to be even more profound in the future.

A broader preparation of the health care system to be able to deal with digitalization is necessary, taking into consideration, training, financial and regulatory needs. Moreover, implementation of monitoring systems to monitor its effects on health system performance, remains important. With the rapidly changing landscape, it is important to develop an overview of the impact of such digital solutions in individual health record systems, including both electronic and personal health records.

#

# Methodology

## OBJECTIVES

A systematic literature review will be performed of the available published peer-reviewed papers. The objectives of the review are:

1. to conduct a systematic review of systematic reviews on the impact of digital solutions in individual health record systems.
2. To develop supporting material for the lecture: “important developments that will shape the health information systems of the future”.
3. To prepare and publish. the manuscript: “The impact of digital solutions in individual health record systems: an overview of systematic reviews”.
4. To summarize the current state of digital advancements research on EHRs and PHRs and provide a comprehensive overview of core technologies that may potentially impact and leverage them.

## SEARCH STRATEGY

A literature search will be performed using MEDLINE (accessed by PubMed), Cochrane (Cochrane Database of Systematic Reviews, Cochrane Central Register of Controlled Trials, Cochrane Methodology Register, Database of Abstracts of Reviews of Effects, Health Technology Assessment, Evidence-Based Practice Center program, National Health Service Economic Evaluation Database), Web of Science, SciELO Citation Index, Current Contents Connect, KCI-Korean Journal Database , Russian Science Citation Index

and Scopus in October 2020. Manual search of references will also be conducted.

**Inclusion criteria**

The following criteria for inclusion will be used to select studies to be included in the review:

1. Population of interest: Search restricted to studies in humans
2. Measures of interest: Systematic reviews on the impact of digital solutions in individual health record systems.
3. Setting: All (community, primary health care, outpatient, inpatient, hospital and emergency, secondary and tertiary health care)
4. Timeframe: from 1^st^ January, 2010 to 10^th^ of October, 2020 (or date of last search run if later)
5. Languages: English, French, Spanish, Italian, and Portuguese).

**Exclusion criteria**

1. Population of interest: non-human studies
2. Other measures of interest
3. Studies that do not fall within the timeframe.
4. Studies in languages other than previously indicated.
5. Not systematic reviews.

We will use a common search strategy and allocate relevant studies to their respective reviews before assessing their risk of bias and extracting data. The following different combinations will be used:

1. Terms for digital solutions:

“health informatics”[tiab] OR “mobile health”[tiab] OR “m-Health”[tiab] OR “mHealth”[tiab] OR “mobile technology”[tiab] OR “mobile phone applications”[tiab] OR “smartphone*”[tiab] OR “app”[ti] OR “apps”[ti] OR “telemedicine”[tiab] OR “telemonitoring”[tiab] OR “tele*”[ti] OR “interoperability” [tiab] OR “Internet”[ti] OR “Web*”[ti] OR “Website*”[ti] OR “health information”[tiab] OR “digital health literacy”[tiab] OR “eHealth literacy”[tiab] OR “online health information”[tiab] OR “on-line health information”[tiab] OR “on-line communit*”[tiab] OR “online communit*”[tiab] OR “social media”[tiab] OR “social network”[tiab] OR “facebook”[tiab] OR “twitter”[tiab] OR “youtube”[tiab] OR “instagram”[tiab] OR “flickr”[tiab] OR “google”[ti] OR “Linkedin”[tiab] OR “blog*”[tiab] OR “wiki*”[tiab] OR “big data”[tiab] OR “open data”[tiab] OR “personalized medicine”[tiab] OR “data mining”[MeSH] OR “wearable*”[tiab] OR “smart health”[tiab] OR “internet of things”[tiab] OR “Wireless Technology”[tiab] OR “cloud”[tiab] OR “bluetooth”[tiab] OR “ehealth”[tiab] OR “e-health”[tiab] OR “digital health”[tiab] OR “information and communication technolog*”[tiab] OR “health information technolog*”[tiab] OR “SMS”[tiab] OR “blockchain”[tiab] OR “data science”[tiab] OR “artificial intelligence”[tiab] OR “machine learning”[tiab] OR “deep learning”[tiab]

1. Terms for Individual health record:

“individual health record*”[tiab] OR “electronic health record*” [MeSH] OR “electronic medical record*”[tiab] OR “electronic personal health record*”[tiab] OR “digital record*”[tiab] OR “health record*”[tiab] OR “personal health record*”[tiab] OR “medical record* system*”[tiab] OR “electronic healthcare record*”[tiab]

1. Terms and filters for “systematic” and “review” identified systematic reviews.

The results of the data searches (Level 1 to 3) will be used to establish the list of reviews on the impact of digital solutions in individual health record systems.

**Table 1. Search strategy for PubMed (a more complete version will be added later).**

| Set | Terms | Number of hits |
| --- | --- | --- |
| 1 | “health informatics”[tiab] OR “mobile health”[tiab] OR “m-Health”[tiab] OR “mHealth”[tiab] OR “mobile technology”[tiab] OR “mobile phone applications”[tiab] OR “smartphone*”[tiab] OR “app”[ti] OR “apps”[ti] OR “telemedicine”[tiab] OR “telemonitoring”[tiab] OR “tele*”[ti] OR “interoperability” [tiab] OR “Internet”[ti] OR “Web*”[ti] OR “Website*”[ti] OR “health information”[tiab] OR “digital health literacy”[tiab] OR “eHealth literacy”[tiab] OR “online health information”[tiab] OR “on-line health information”[tiab] OR “on-line communit*”[tiab] OR “online communit*”[tiab] OR “social media”[tiab] OR “social network”[tiab] OR “facebook”[tiab] OR “twitter”[tiab] OR “youtube”[tiab] OR “instagram”[tiab] OR “flickr”[tiab] OR “google”[ti] OR “Linkedin”[tiab] OR “blog*”[tiab] OR “wiki*”[tiab] OR “big data”[tiab] OR “open data”[tiab] OR “personalized medicine”[tiab] OR “data mining”[MeSH] OR “wearable*”[tiab] OR “smart health”[tiab] OR “internet of things”[tiab] OR “Wireless Technology”[tiab] OR “cloud”[tiab] OR “bluetooth”[tiab] OR “ehealth”[tiab] OR “e-health”[tiab] OR “digital health”[tiab] OR “information and communication technolog*”[tiab] OR “health information technolog*”[tiab] OR “SMS”[tiab] OR “blockchain”[tiab] OR “data science”[tiab] OR “artificial intelligence”[tiab] OR “machine learning”[tiab] OR “deep learning”[tiab] | 274,721 |
| 2 | “individual health record*”[tiab] OR “electronic health record*” [MeSH] OR “electronic medical record*”[tiab] OR “electronic personal health record*”[tiab] OR “digital record*”[tiab] OR “health record*”[tiab] OR “personal health record*”[tiab] OR “medical record* system*” [tiab] | 48,472 |
| 3 | 1+2 | 10,009 |
| 4 | 1+2+3[tiab] filtered by period (1/1/2020- to date) and humans | 862 |
| 5 | 1+2+3[ti] filtered by period (1/1/2020- to date) and humans | 229 |
|  | (same search performed in Portuguese, Italian, French and Spanish) |  |

Tips on the Search Strategy

Handsearching of journals: 1. It will be documented using the full title of the journal and the ﬁrst and latest years searched. The abstracts may contain sufﬁcient detail to enable relevance screening.

INFORMATION SOURCES

**Electronic databases and search strategy**

A literature search will be performed using MEDLINE (accessed by PubMed), Cochrane (Cochrane Database of Systematic Reviews, Cochrane Central Register of Controlled Trials, Cochrane Methodology Register, Database of Abstracts of Reviews of Effects, Health Technology Assessment, Evidence-Based Practice Center program, National Health Service Economic Evaluation Database), Web of Science, and Scopus in October 2020. Manual search of references will also be conducted.

**Additional data**

Additionally, we will hand search the reference list of relevant studies retrieved from the electronic database search to identify additional studies. Furthermore, data may be obtained by directly contacting researchers if needed. We can prepare a form for collaborators to ﬁll in with information.

## PROTOCOL FOR DATA EXTRACTION

### Data Extraction and Quality Assessment

Data extraction will be conducted following standardized criteria, and results will be reviewed by at least two senior researchers.

Studies will be evaluated using the Measurement Tool to Assess Systematic Reviews (AMSTAR) checklist for assessing methodological quality. Several standards were developed to minimize bias and subjectivity in a review process. Among these are those established by the [Cochrane](https://www.cochrane.org/)  initiatives to conduct systematic reviews as well as the Preferred Reporting Items for Systematic Reviews and Meta-Analyses (PRISMA) statement and the methodological considerations when using existing systematic reviews^2^. We will apply many of these standards, setting a priori the criteria to search for, select, and extract the data, documenting each step of the process.

The reliability in the data extraction process will be tested by having it conducted independently by at least two evaluators with a sample of the data sources. Reliability and inter-evaluator consistency will be documented. The forms for the capture of data will specify the data and variables of interest, with instructions on how data extraction should be performed. The following data will be extracted: journal, publication year, databases searched, time period, objective, number of studies, study design, area, outcomes, opportunities, solutions, main results, and main challenges for implementation.

**Table 2. Data items for extraction**

| **ﬁeld_name** | **label** | **explanation** |
| --- | --- | --- |
| Title | title | Title of the study |
| Journal | Journal | Journal / source of publication |
| year | Publication year | Year of publication |
| DB | Database source | Source from which this study was obtained |
| Solution | Solution type | Type of intervention |
| No | Number of studies | Number of studies included in the review. |
| Design | Study design | Type of study which complies with inclusion criteria (a systematic review). |
| Area | Area | Technological area (i.e., artificial intelligence, blockchain,etc.) |
| Opportunity | Opportunity | Opportunities of the technological tool |
| Objective | Objective | Objective of the review |
| Results | Results | Main results of the review |
| Challenges | Challenges | Barriers for implementation |

### Data Management and Storage

Information on data sources (i.e. lists of references) will be uploaded onto the database platform through *.bib ﬁles (Bibtex). The following reference manager software wiil be used: Endnote, Zotero and Paperpile to export and manage the references to the bib ﬁle, which can then be ‘dragged and dropped’ into the platform. When adding new data sources to the database. We will register how they were identiﬁed (if searching databases, by cross-referencing, by experts, etc), so the PRISMA diagram below (needed for publication and communication of the results) can be ﬁlled appropriately. In the case of database search, it is important to take note of the search terms and number of hits obtained (so a table like **Table 1**, shown before, can be properly ﬁlled), as well as indicate how many sources were excluded through screening of abstracts/titles (**Step 1** in the diagram), and how many were excluded after full text inspection (**Step 2** in the diagram). Search logs and results will be documented. The selection process will take place in two steps. In the first step, two reviewers will review the titles and abstracts obtained from the electronic searches and apply the inclusion/exclusion criteria. The second selection step will be based on the full-text review of the articles retrieved in the first step. Data from the full-text selected papers will be extracted using a standard extraction form to ensure we gather all relevant data systematically and stored in MS Excel compatible format. Data extraction will be carried out by two reviewers.


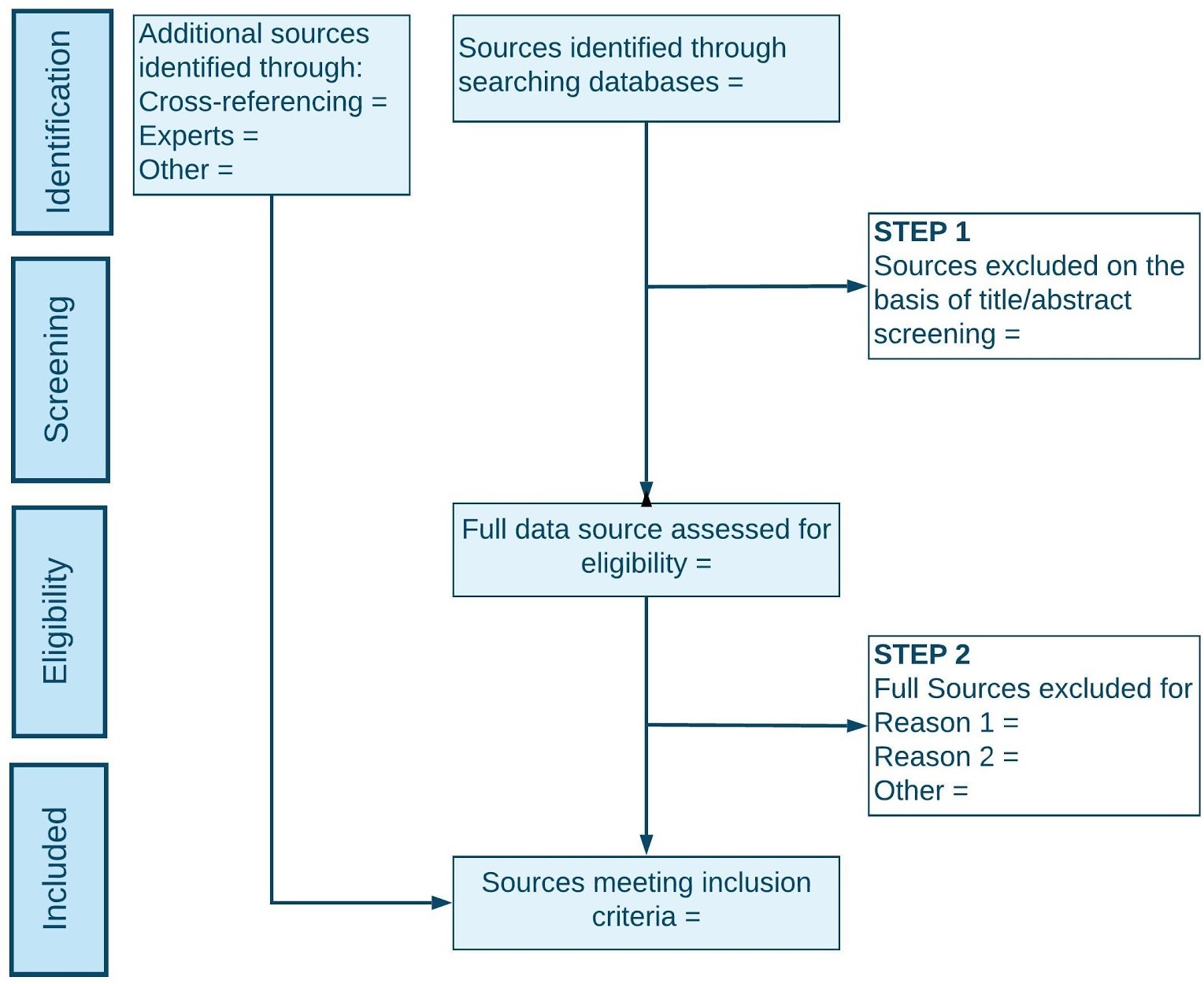


**Figure 1. Prisma Diagram**

# References

1. World Health Organization. Health Technology. What is health technology? Published 2019. Accessed September 16, 2020. https://www.who.int/health-technology-assessment/about/healthtechnology/en/.

2. Whitlock EP, Lin JS, Chou R, Shekelle P, Robinson KA. Using existing systematic reviews in complex systematic reviews. *Ann Intern Med*. 2008;148(10):776-782. doi:10.7326/0003-4819-148-10-200805200-00010

# Annex 1: Search Strategy

Search strategy for the “Review of Systematic Reviews on the Impact of Digital Solutions in Individual Health Record Systems” is shown below. The chosen option is shaded in pink.

**Pubmed Search**

| **Ref.** | **Search terms related to Digital Solutions** | **No of hits** | **Filters** |
| --- | --- | --- | --- |
| N#1 | "mHealth"[tiab] | 5,522 |  |
| N#2 | m-Health”[tiab] | 569 |  |
| N#3 | m-Health[tiab] or mHealth[tiab] | 5,557 |  |
| N#4 | mobile health[MeSH Terms] | 19,163 |  |
| N#5 | m-Health[tiab] or mHealth[tiab] OR mobile health[MeSH Terms] | 22,740 |  |
| N#6 | health informatics[tiab] | 1,749 |  |
| N#7 | health information technologies[MeSH Terms] | 254,759 |  |
| N#8 | ehealth[MeSH Terms] | 19,163 |  |
| N#9 | health information technologies[MeSH Terms] OR ehealth[MeSH Terms] | 271,498 |  |
| N#10 | mobile phone[MeSH Terms] | 9,118 |  |
| N#11 | social media[MeSH Terms] | 8,359 |  |
| N#12 | social network[MeSH Terms] | 3,983 |  |
| N#13 | social network[MeSH Terms] OR social media[MeSH Terms] | 11,546 |  |
| N#14 | internet[MeSH Terms] | 47,965 |  |
| N#15 | ai artificial intelligence[MeSH Terms] | 64,876 |  |
| N#16 | machine learning[MeSH Terms] | 20,561 |  |
| N#17 | “health informatics”[tiab] OR “mobile health”[tiab] OR “m-Health”[tiab] OR “mHealth”[tiab] OR “mobile technology”[tiab] OR “mobile phone applications”[tiab] OR “smartphone*”[tiab] OR “app”[ti] OR “apps”[ti] OR “telemedicine”[tiab] OR “telemonitoring”[tiab] OR “tele*”[ti] OR “interoperability” [tiab] OR “Internet”[ti] OR “Web*”[ti] OR “Website*”[ti] OR “health information”[tiab] OR “digital health literacy”[tiab] OR “eHealth literacy”[tiab] OR “online health information”[tiab] OR “on-line health information”[tiab] OR “on-line communit*”[tiab] OR “online communit*”[tiab] OR “social media”[tiab] OR “social network”[tiab] OR “facebook”[tiab] OR “twitter”[tiab] OR “youtube”[tiab] OR “instagram”[tiab] OR “flickr”[tiab] OR “google”[ti] OR “Linkedin”[tiab] OR “blog*”[tiab] OR “wiki*”[tiab] OR “big data”[tiab] OR “open data”[tiab] OR “personalized medicine”[tiab] OR “data mining”[MeSH] OR “wearable*”[tiab] OR “smart health”[tiab] OR “internet of things”[tiab] OR “Wireless Technology”[tiab] OR “cloud”[tiab] OR “bluetooth”[tiab] OR “ehealth”[tiab] OR “e-health”[tiab] OR “digital health”[tiab] OR “information and communication technolog*”[tiab] OR “health information technolog*”[tiab] OR “SMS”[tiab] OR “blockchain”[tiab] OR “data science”[tiab] OR “artificial intelligence”[tiab] OR “machine learning”[tiab] OR “deep learning”[tiab] | 206,532 |  |
| N#18 | (machine learning[MeSH Terms]) OR (ai artificial intelligence[MeSH Terms])) OR (internet[MeSH Terms])) OR (social network[MeSH Terms])) OR (social media[MeSH Terms])) OR (mobile phone[MeSH Terms])) OR (health information technologies[MeSH Terms] OR ehealth[MeSH Terms])) OR (ehealth[MeSH Terms])) OR (health information technologies[MeSH Terms])) OR (mobile health[MeSH Terms]) | 355,545 |  |
| N#19 | N#17 OR N#18 | 485,738 | Filters: **from 2010/1/1 - 2020/10/6** |
| N#20 | N#17 OR (health information technologies[MeSH Terms]) | 432,432 | Filters: **from 2010/1/1 - 2020/10/6** |
| N#21 | N#20 | 44,227 | Filters: Meta-Analysis, Review, Systematic Review, |
| **Ref.** | **Search terms related to electronic /individual /personal Health Record Systems** | **No of hits** | **Filters** |
| P#1 | electronic health records[MeSH Terms] | 20,512 |  |
| P#2 | electronic health record[MeSH Terms] | 20,512 |  |
| P#3 | personal electronic health records[MeSH Terms] | 10,246 |  |
| P#4 | health records, personal[MeSH Terms] | 1,903 |  |
| P#5 | P#3 OR P#4 | 11,401 |  |
| P#6 | P#1 OR P#5 | 21,622 |  |
| P#7 | individual health record*[tiab] | 12 |  |
| P#8 | electronic medical record*[tiab] | 14,910 |  |
| P#9 | electronic personal health record*[tiab] | 99 |  |
| P#10 | digital record*[tiab] | 355 |  |
| P#11 | health record*[tiab] | 18,859 |  |
| P#12 | personal health record*[tiab] | 885 |  |
| P#13 | medical record* system*[tiab] | 977 |  |
| P#14 | P#7 OR P#8 OR P#9 OR P#10 OR P#11 OR P#12 OR P#13 | 33,352 |  |
| P#15 | P#14 OR P#3  (individual health record*[tiab] OR (electronic medical record*[tiab] OR (electronic personal health record*[tiab] OR (digital record*[tiab] OR (health record*[tiab] OR (personal health record*[tiab] OR (medical record* system*[tiab] OR (electronic health record*[MeSH] OR (personal health records[MeSH Terms]) | 43,489 | Filters: 2010/1/1:2020/10/6[pdat])) |
| P#16 | P#15 AND review*[tiab] | 6,855 | = |
| P#17 | P#15 | 2,612 | Filters: Meta-Analysis, Review, Systematic Review |
| P#18 | P#15 ( Filters applied: Humans, English, French, Italian, Portuguese, Spanish, from 2010/1/1 - 2020/10/6.) | 1,930 | Filters: Humans, English, French, Italian, Portuguese, Spanish  Filters: Meta-Analysis, Review, Systematic Review |
| P#19 | P#18 AND record*[tiab] | 1,577 | = |
| P#20 | P#18 AND record*[ti] | 390 | = |
| P#21 | P#18 AND review*[tiab] | 1,228 | Filters applied: Meta-Analysis, Review, Systematic Review, Humans, English, French, Italian, Portuguese, Spanish, from 2010/1/1 - 2020/10/6. |

**Scopus Search**

*2010 -2020*

| **Ref.** | **Search terms related to Individual Health Record Systems and reviews** | **No of hits** | **Filters** |
| --- | --- | --- | --- |
| P#1 | TITLE-ABS ( individual AND health AND record* ) OR TITLE-ABS ( electronic AND medical AND record* ) OR TITLE-ABS ( electronic AND personal AND health AND record* ) OR TITLE-ABS ( digital AND record* ) OR TITLE-ABS ( health AND record* ) OR TITLE-ABS ( personal AND health AND record* ) OR TITLE-ABS ( medical AND record* AND system* ) OR TITLE-ABS ( electronic AND health AND record* ) | 267,721 |  |
| P#2 | TITLE ( record* ) AND TITLE-ABS ( review ) | 6,800 |  |
| P#3 | TITLE ( record* ) AND TITLE-ABS ( review ) AND ( LIMIT-TO ( EXACTKEYWORD , "Medical Record" ) OR LIMIT-TO ( EXACTKEYWORD , "Electronic Health Records" ) ) | 1,209 | AND ( LIMIT-TO ( LANGUAGE , "English" ) OR LIMIT-TO ( LANGUAGE , "Spanish" ) OR LIMIT-TO ( LANGUAGE , "French" ) OR LIMIT-TO ( LANGUAGE , "Portuguese" ) OR LIMIT-TO ( LANGUAGE , "Italian" ) ) AND ( LIMIT-TO ( PUBYEAR , 2020 -2010) |

| **Ref.** | **Search terms related to Individual Health Record Systems and Reviews** | **No of hits** | **Filters** |
| --- | --- | --- | --- |
| P#1 | (TI=record OR AB=RECORD) AND (TI=REVIEW OR AB=REVIEW) | [140,281](https://apps.webofknowledge.com/summary.do?product=UA&doc=1&qid=5&SID=D4eCFbfksBhY6tLLIxG&search_mode=AdvancedSearch&update_back2search_link_param=yes) |  |
| P#2 | (TI=record*) AND (TI=REVIEW*) | [1,521](https://apps.webofknowledge.com/summary.do?product=UA&doc=1&qid=9&SID=D4eCFbfksBhY6tLLIxG&search_mode=AdvancedSearch&update_back2search_link_param=yes) |  |
| P#3 | (TI=record OR TI=RECORDS) AND (TI=REVIEW) | 1,456 |  |
| P#4 | (TI=record OR TI=RECORDS) AND (TI=REVIEW) | 790 | Refined by: DOCUMENT TYPES: ( REVIEW ) |
| P#5 | (TI=record OR TI=RECORDS) AND (TI=REVIEW OR AB=REVIEW) | 5,081 | Refined by: DOCUMENT TYPES: ( REVIEW ) Languages: Spanish ,English, French, Italian, Portuguese |
| P#6 | (TI=record OR TI=RECORDS) AND (TI=REVIEW OR AB=REVIEW) | 1,198 | Refined by: DOCUMENT TYPES: ( REVIEW ) Languages: Spanish ,English, French, Italian, Portuguese |
| P#7 | (TI=record OR TI=RECORDS) AND (TI=REVIEW OR AB=REVIEW) | 630 | As above plus refine by research domain: healthcare sciences services , medical informatics , information science library science, computer science, science technology other topics |

**Web of Science Search**

*Databases= WOS, CCC, DIIDW, KJD, MEDLINE Timespan=2010-2020*

MEDLINE® ; KCI-Korean Journal Database ; Russian Science Citation Index ; SciELO Citation Index ; Current Contents Connect

**Cochrane Library**

| **Ref.** | **Search terms related to Individual Health Record Systems and Reviews** | **No of hits** | **Filters** |
| --- | --- | --- | --- |
| P#1 | "medical record review studies" | [1](https://apps.webofknowledge.com/summary.do?product=UA&doc=1&qid=5&SID=D4eCFbfksBhY6tLLIxG&search_mode=AdvancedSearch&update_back2search_link_param=yes) | keyword |
| P#2 | "medical record review study" | 0 | keyword |
| P#3 | "individual medical record" | 0 | keyword |
| P#4 | "electronic health care record" | 0 | keyword |
| P#5 | "electronic health record" | 1 | Mesh Term |
| P#6 | "electronic healthcare records" | 0 | keyword |
| P#7 | "personal health information" | 0 | keyword |
| P#8 | "medical record review studies" | 322 | Title abstract and keyword |

# Annex II: Prisma Flowchart

#
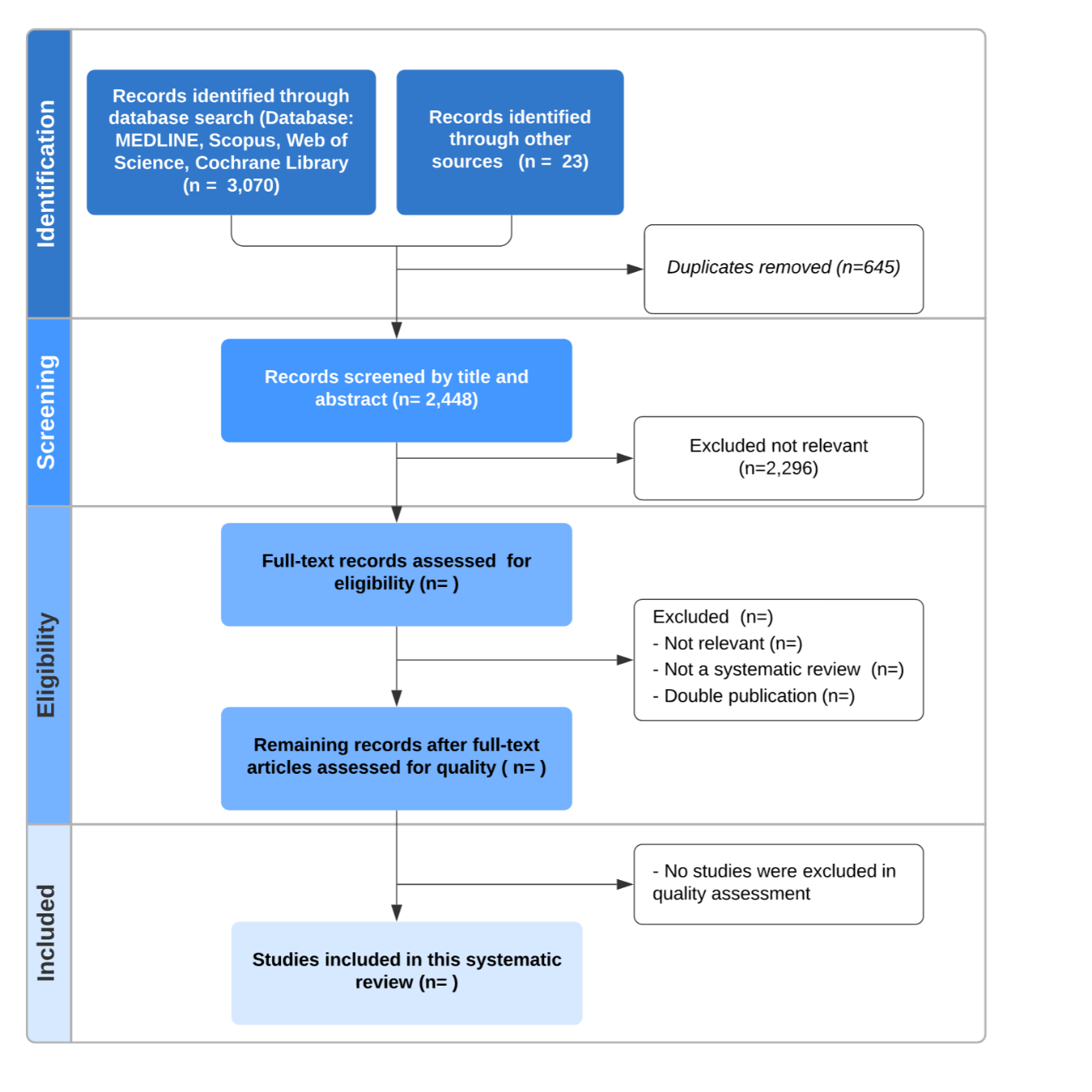

Supplement: Supplementary file 1 [file mmc1.docx]
