# Supplementary material for: Technological progress in electronic health record system optimization: Systematic review of systematic literature reviews
Source: Int J Med Inform. 2021 Aug;152:104507. doi: 10.1016/j.ijmedinf.2021.104507 (PMC8223493; doi:10.1016/j.ijmedinf.2021.104507)
Supplement: Supplementary file 2 [file mmc2.docx]

**Appendix A. Search strategy**

| Database | Search Terms | No. of hits |
| --- | --- | --- |
| PubMed | individual health record*[tiab] OR (electronic medical record*[tiab] OR (electronic personal health record*[tiab] OR (digital record*[tiab] OR (health record*[tiab] OR (personal health record*[tiab] OR (medical record* system*[tiab] OR (electronic health record*[MeSH] OR (personal health records[MeSH Terms])  **Filters applied**: Meta-Analysis, Review, Systematic Review, Humans, English, French, Italian, Portuguese, Spanish, from 2010/1/1 - 2020/10/6. | 1,228 |
|  | ((individual health record*[tiab] OR (electronic medical record*[tiab] OR (electronic personal health record*[tiab] OR (digital record*[tiab] OR (health record*[tiab] OR (personal health record*[tiab] OR (medical record* system*[tiab] OR (electronic health record*[MeSH] OR (personal health records[MeSH Terms])) AND (Digital[Title/Abstract])  **Filters applied**: Meta-Analysis, Review, Systematic Review, Humans, English, French, Italian, Portuguese, Spanish, from 2010/1/1 - 2020/10/6. | 153 |
| Scopus | TITLE ( record* ) AND TITLE-ABS ( review ) AND ( LIMIT-TO ( EXACTKEYWORD , "Medical Record" ) OR LIMIT-TO ( EXACTKEYWORD , "Electronic Health Records" ) )  AND ( LIMIT-TO ( LANGUAGE , "English" ) OR LIMIT-TO ( LANGUAGE , "Spanish" ) OR LIMIT-TO ( LANGUAGE , "French" ) OR LIMIT-TO ( LANGUAGE , "Portuguese" ) OR LIMIT-TO ( LANGUAGE , "Italian" ) ) AND ( LIMIT-TO ( PUBYEAR , 2020 -2010) | 1,209 |
| Web of Science | ((TI=record OR TI=RECORDS) AND (TI=REVIEW OR AB=REVIEW)) AND LANGUAGE: (English)  **Refined by:** WEB OF SCIENCE CATEGORIES: (HEALTH CARE SCIENCES SERVICES OR MEDICAL INFORMATICS OR COMPUTER SCIENCE INFORMATION SYSTEMS OR COMPUTER SCIENCE INTERDISCIPLINARY APPLICATIONS)  **Timespan**: 2010-2020.  **Indexes:** SCI-EXPANDED, SSCI, A&HCI, CPCI-S, CPCI-SSH, BKCI-S, BKCI-SSH, ESCI, CCR-EXPANDED, IC. | 630 |
| Cochrane Database of Systematic Reviews | "electronic health record" Mesh Term | 1 |
