# Supplementary material for: Technological progress in electronic health record system optimization: Systematic review of systematic literature reviews
Source: Int J Med Inform. 2021 Aug;152:104507. doi: 10.1016/j.ijmedinf.2021.104507 (PMC8223493; doi:10.1016/j.ijmedinf.2021.104507)
Supplement: Supplementary file 3 [file mmc3.docx]

**Appendix B. Excluded articles by full-text review and reasons for exclusion n=49**

Not relevant to research question n= 31

Full-text not available n= 2

Literature search strategy very limited in time n= 2

Not a systematic review n= 14

| **Author(s), Year** | **Ref.** | **Reason for Exclusion** |
| --- | --- | --- |
| Ajami, 2016 | [1] | Full-text not available |
| Archer et al., 2011 | [2] | Not relevant to research question |
| Belletti, Zacker and Mullins, 2010 | [3] | Not a systematic review |
| Bouayad, et al, 2017 | [4] | Not relevant to research question |
| Crampton, et al., 2016 | [5] | Not relevant to research question |
| Cheeseman, 2011 | [6] | Not a systematic review |
| Demiris et al., 2019 | [7] | Not relevant to research question |
| Demiris, et al., 2015 | [8] | Not relevant to research question |
| de Mesquita and Edwards, 2020 | [9] | Not relevant to research question |
| Dinh-Le et al., 2019 | [10] | Not a systematic review |
| Dobrow et al., 2019 | [11] | Not relevant to research question |
| Dreisbach et al., 2019 | [12] | Not relevant to research question |
| Ford et al., 2016 | [13] | Literature search strategy for a limited timeframe |
| Haddad, Souza and Cecatti, 2019 | [14] | Not relevant to research question |
| Hawthorne and Richards, 2017 | [15] | Not a systematic review |
| Health Quality Ontario, 2013 | [16] | Not relevant to research question |
| Hendrickson, Melton and Pitt, 2019 | [17] | Not a systematic review |
| Hwang et al., 2010 | [18] | Not a systematic review |
| Hypponen et al., 2014 | [19] | Not a systematic review (protocol of a SLR) |
| Idri et al., 2018 | [20] | Not relevant to research question |
| Johansen et al., 2014 | [21] | Not relevant to research question |
| Jones et al., 2014 | [22] | Not relevant to research question |
| Jones et al., 2018 | [23] | Not relevant to research question |
| Lai et al., 2017 | [24] | Not a systematic review |
| Mangesius et al., 2017 | [25] | Not relevant to research question |
| Mars and Scott, 2016 | [26] | Not relevant to research question |
| Martin et al., 2018 | [27] | Not relevant to research question |
| Mehta and Pandit, 2018 | [28] | Not relevant to research question |
| Menachemi and Collum, 2011 | [29] | Not relevant to research question |
| Menachemi et al., 2018 | [30] | Not relevant to research question |
| Mukund Bahadur and Murray, 2010 | [31] | Not relevant to research question |
| Névéol and Zweigenbaum, 2015 | [32] | Literature search strategy for a limited timeframe |
| Payrovnaziri et al., 2020 | [33] | Not relevant to research question |
| Reeder et al., 2013 | [34] | Not relevant to research question |
| Renshaw et al., 2018 | [35] | Not a systematic review |
| Rijo et al., 2015 | [36] | Not a systematic review |
| Roehrs et al., 2017 | [37] | Not relevant to research question |
| Ross, Wei and Ohno-Machado, 2014 | [38] | Not a systematic review |
| Roth et al., 2018 | [39] | Not a systematic review |
| Saha et al., 2019 | [40] | Not a systematic review |
| Sivanathan, Ritchie and Lim, 2017 | [41] | Not relevant to research question |
| Studeny and Coustasse, 2014 | [42] | Not relevant to research question |
| Tsirintani, 2016 | [43] | Full-text not available |
| Vimalananda et al., 2015 | [44] | Not relevant to research question |
| Voruganti et al., 2017 | [45] | Not relevant to research question |
| White and Roudsari, 2011 | [46] | Not relevant to research question |
| Younas et al., 2017 | [47] | Not relevant to research question |
| Zieth et al., 2014 | [48] | Not relevant to research question |
| Zhang and Zhang, 2016 | [49] | Not a systematic review |

**References**

1. Ajami S. Use of speech-to-text technology for documentation by healthcare providers. Natl Med J India. 2016;29: 148–152.

2. Archer N, Fevrier-Thomas U, Lokker C, McKibbon KA, Straus SE. Personal health records: A scoping review. J Am Med Inform Assoc. 2011;18: 515–522.

3. Belletti D, Zacker C, Mullins CD. Perspectives on electronic medical records adoption: electronic medical records (EMR) in outcomes research. Patient Relat Outcome Meas. 2010;1: 29–37.

4. Bouayad L, Ialynytchev A, Padmanabhan B. Patient health record systems scope and functionalities: Literature review and future directions. J Med Internet Res. 2017;19: e388.

5. Crampton NH, Reis S, Shachak A. Computers in the clinical encounter: a scoping review and thematic analysis. J Am Med Inform Assoc. 2016;23: 654–665.

6. Cheeseman SE. Are you prepared for the digital era? Neonatal Netw. 2011;30: 263–266.

7. Demiris G, Iribarren SJ, Sward K, Lee S, Yang R. Patient generated health data use in clinical practice: A systematic review. Nurs Outlook. 2019;67: 311–330.

8. Demiris G, Kneale L. Informatics Systems and Tools to Facilitate Patient-centered Care Coordination. Yearb Med Inform. 2015;10: 15–21.

9. de Mesquita RC, Edwards I. SYSTEMATIC LITERATURE REVIEW OF MY HEALTH RECORD SYSTEM. ASIA PACIFIC JOURNAL OF HEALTH MANAGEMENT. 2020;15: 14–25.

10. Dinh-Le C, Chuang R, Chokshi S, Mann D. Wearable Health Technology and Electronic Health Record Integration: Scoping Review and Future Directions. JMIR Mhealth Uhealth. 2019;7: e12861.

11. Dobrow MJ, Bytautas JP, Tharmalingam S, Hagens S. Interoperable Electronic Health Records and Health Information Exchanges: Systematic Review. JMIR MEDICAL INFORMATICS. 2019;7: 172–181.

12. Dreisbach C, Koleck TA, Bourne PE, Bakken S. A systematic review of natural language processing and text mining of symptoms from electronic patient-authored text data. Int J Med Inform. 2019;125: 37–46.

13. Ford E, Carroll JA, Smith HE, Scott D, Cassell JA. Extracting information from the text of electronic medical records to improve case detection: A systematic review. J Am Med Inform Assoc. 2016;23: 1007–1015.

14. Haddad SM, Souza RT, Cecatti JG. Mobile technology in health (mHealth) and antenatal care-Searching for apps and available solutions: A systematic review. Int J Med Inform. 2019;127: 1–8.

15. Hawthorne KH, Richards L. Personal health records: a new type of electronic medical record. RECORDS MANAGEMENT JOURNAL. 2017;27: 286–301.

16. Health Quality Ontario. Electronic tools for health information exchange: an evidence-based analysis. Ont Health Technol Assess Ser. 2013;13: 1–76.

17. Hendrickson MA, Melton GB, Pitt MB. The Review of Systems, the Electronic Health Record, and Billing. JAMA - Journal of the American Medical Association. 2019;322: 115–116.

18. Hwang KH, Chung K-I, Chung M-A, Choi D. Review of semantically interoperable electronic health records for ubiquitous healthcare. Healthc Inform Res. 2010;16: 1–5.

19. Hypponen H, Saranto K, Vuokko R, Makela-Bengs P, Doupi P, Lindqvist M, et al. Impacts of structuring the electronic health record: A systematic review protocol and results of previous reviews. Int J Med Inform. 2014;83: 159–169.

20. Idri A, Benhar H, Fernández-Alemán JL, Kadi I. A systematic map of medical data preprocessing in knowledge discovery. Comput Methods Programs Biomed. 2018;162: 69–85.

21. Johansen, Monika, A, Henriksen, Eva. The evolution of personal health records and their role for self-management: a literature review. In: Pape-Haugaard L., Seroussi Brigitte B., Saka O., Lovis C., Hasman A., Andersen S.K., editors. 25th European Medical Informatics Conference (MIE). Istanbul, TURKEY: IOS Press; 2014. pp. 458–462.

22. Jones LM, Veinot TCE, Pressler SJ, Seng JS, McCall AM, Fernandez D, et al. Internet health information seeking (IHIS): an integrative review of the literature. West J Nurs Res. 2014;36: 1376–1377.

23. Jones KH, Daniels H, Heys S, Ford DV. Challenges and Potential Opportunities of Mobile Phone Call Detail Records in Health Research: Review. JMIR Mhealth Uhealth. 2018;6: e161.

24. Lai AM, Hsueh P-YS, Choi YK, Austin RR. Present and Future Trends in Consumer Health Informatics and Patient-Generated Health Data. Yearb Med Inform. 2017;26: 152–159.

25. Mangesius P, Saboor S, Healy T, Schabetsberger T. Dynamic Creation of Patient Summaries: A CDA and IHE XDS Based Approach for Regional EHRs. Stud Health Technol Inform. 2017;245: 170–173.

26. Mars M, Scott RE. WhatsApp in Clinical Practice: A Literature Review. Stud Health Technol Inform. 2016;231: 82–90.

27. Martin TJ, Ranney ML, Dorroh J, Asselin N, Sarkar IN. Health Information Exchange in Emergency Medical Services. Appl Clin Inform. 2018;9: 884–891.

28. Mehta N, Pandit A. Concurrence of big data analytics and healthcare: A systematic review. Int J Med Inform. 2018;114: 57–65.

29. Menachemi N, Collum TH. Benefits and drawbacks of electronic health record systems. Risk Manag Healthc Policy. 2011;4: 47–55.

30. Menachemi, Nir, Rahurkar, Saurabh, Harle, Christopher, et al. The benefits of health information exchange: an updated systematic review. J Am Med Inform Assoc. 2018;25: 1259–1265.

31. Mukund Bahadur K-C, Murray PJ. Cell phone short messaging service (SMS) for HIV/AIDS in South Africa: a literature review. Stud Health Technol Inform. 2010;160: 530–534.

32. Névéol A, Zweigenbaum P. Clinical Natural Language Processing in 2014: Foundational Methods Supporting Efficient Healthcare. Yearb Med Inform. 2015;10: 194–198.

33. Payrovnaziri SN, Chen Z, Rengifo-Moreno P, Miller T, Bian J, Chen JH, et al. Explainable artificial intelligence models using real-world electronic health record data: a systematic scoping review. J Am Med Inform Assoc. 2020;27: 1173–1185.

34. Reeder B, Meyer E, Lazar A, Chaudhuri S, Thompson HJ, Demiris G. Framing the evidence for health smart homes and home-based consumer health technologies as a public health intervention for independent aging: a systematic review. Int J Med Inform. 2013;82: 565–579.

35. Renshaw AA, Mena-Allauca M, Gould EW, Sirintrapun SJ. Synoptic Reporting: Evidence-Based Review and Future Directions. JCO Clin Cancer Inform. 2018;2: 1–9.

36. Rijo R, Martinho R, Pereira L, Silva C. Text Mining Applied to Electronic Medical Records: A Literature Review. Int J E-Health Med Commun. 2015;6: 1–18.

37. Roehrs A, da Costa CA, Righi R da R, Farias de Oliveira KS. Personal health records: A systematic literature review. J Med Internet Res. 2017;19: e13.

38. Ross MK, Wei W, Ohno-Machado L. “Big data” and the electronic health record. Yearb Med Inform. 2014;9: 97–104.

39. Roth JA, Battegay M, Juchler F, Vogt JE, Widmer AF. Introduction to Machine Learning in Digital Healthcare Epidemiology. Infect Control Hosp Epidemiol. 2018;39: 1457–1462.

40. Saha A, Amin R, Kunal S, Vollala S, Dwivedi SK. Review on “Blockchain technology based medical healthcare system with privacy issues.” Secur Priv. 2019;2. doi:10.1002/spy2.83

41. Sivanathan A, Ritchie JM, Lim T. A novel design engineering review system with searchable content: knowledge engineering via real-time multimodal recording. J Eng Des. 2017;28: 681–708.

42. Studeny J, Coustasse A. Personal health records: is rapid adoption hindering interoperability? Perspect Health Inf Manag. 2014;11: 1e.

43. Tsirintani M. Mobile technologies in healthcare and electronic health records. In: Mantas J., Househ M.S., Hasman A., Gallos P., Kolokathi A., editors. 14th Annual International Conference on Informatics, Management, and Technology in Healthcare (ICIMTH). Athens, GREECE: IOS Press; 2016. pp. 249–252.

44. Vimalananda VG, Gupte G, Seraj SM, Orlander J, Berlowitz D, Fincke BG, et al. Electronic consultations (e-consults) to improve access to specialty care: a systematic review and narrative synthesis. J Telemed Telecare. 2015;21: 323–330.

45. Voruganti T, Grunfeld E, Makuwaza T, Bender JL. Web-Based Tools for Text-Based Patient-Provider Communication in Chronic Conditions: Scoping Review. J Med Internet Res. 2017;19: e366.

46. White P, Roudsari A. Use of ontologies for monitoring electronic health records for compliance with clinical practice guidelines. IOS Press; 2011.

47. Younas A, Malik MSA, Khalil-Ur-Rehman, Shahid R. A detailed study on temporal data visualization techniques in electronic health records. Institute of Electrical and Electronics Engineers Inc.; 2017.

48. Zieth CR, Chia LR, Roberts MS, Fischer GS, Clark S, Weimer M, et al. The evolution, use, and effects of integrated personal health records: A narrative review. Electronic Journal of Health Informatics. 2014;8.

49. Zhang X-Y, Zhang P. Recent perspectives of electronic medical record systems (Review). Exp Ther Med. 2016;11: 2083–2085.
