# Supplementary material for: Technological progress in electronic health record system optimization: Systematic review of systematic literature reviews
Source: Int J Med Inform. 2021 Aug;152:104507. doi: 10.1016/j.ijmedinf.2021.104507 (PMC8223493; doi:10.1016/j.ijmedinf.2021.104507)
Supplement: Supplementary file 4 [file mmc4.docx]

**Appendix C. Quality assessment ratings of systematic reviews included in digital tools review**

| Author, year | Reference | Quality assessment questions | | | | | | | | | | Score summary | | | | | Q11 - Funding source for the systematic review is reported? |
| --- | --- | --- | --- | --- | --- | --- | --- | --- | --- | --- | --- | --- | --- | --- | --- | --- | --- |
|  |  | Q1 - Did the review clearly show the purpose of the research? | Q2 - Did the review adequately describe the literature review, background? | Q3 - Did the review authors use a comprehensive search strategy? | Q4 - Did the review authors perform study selection in duplicate? | Q5 - Did the review authors perform data extraction in duplicate? | Q6 - Did the review authors provide a list of excluded studies and justify? | Q7 - Did the review authors describe the included studies detail? | Q8 - Was the scientific quality of the included studies assessed? | Q9 - Was there a satisfactory explanation for heterogenicity? | Q10 - Did the review authors report any potential conflict of interest? | | No | Yes | Yes, partial | Unknown |  |
| Blackley et al., 2019 | [1] | y | y | py | y | unk | n | y | n | y | y | | 2 | 6 | 1 | 1 | Yes |
| Chukwu et al., 2020 | [2] | y | y | y | unk | unk | n | y | y | y | n | | 2 | 6 | 0 | 2 | No |
| Dainton et al., 2017 | [3] | y | y | y | y | y | n | y | py | y | y | | 1 | 8 | 1 | 0 | Yes |
| Dubovitskaya et al., 2020 | [4] | y | y | py | y | y | n | y | n | y | y | | 2 | 7 | 1 | 0 | Yes |
| Hasselgren et al., 2020 | [5] | y | y | y | y | y | n | y | y | y | y | | 1 | 9 | 0 | 0 | Yes |
| Hussien et al, 2019 | [6] | y | y | y | unk | unk | n | y | n | y | y | | 2 | 6 | 0 | 2 | Yes |
| Juhn et al., 2020 | [7] | y | y | y | n | n | n | y | n | y | n | | 5 | 5 | 0 | 0 | No |
| Koleck et al., 2019 | [8] | y | y | y | y | y | n | y | y | y | y | | 1 | 9 | 0 | 0 | Yes |
| Kreimeyer et al., 2017 | [9] | y | y | y | y | y | py | y | n | y | y | | 1 | 8 | 1 | 0 | Yes |
| Kumah-Crystal et al., 2018 | [10] | y | y | py | y | y | n | y | n | y | y | | 2 | 7 | 1 | 0 | No |
| Mayer et al., 2020 | [11] | y | y | y | y | y | n | y | y | y | y | | 1 | 9 | 0 | 0 | Yes |
| Mazlan et al., 2020 | [12] | y | y | y | unk | unk | n | y | n | y | n | | 3 | 5 | 0 | 2 | Yes |
| Meystre et al., 2010 | [13] | y | y | y | n | n | n | y | n | y | y | | 4 | 6 | 0 | 0 | Yes |
| Mishra et al., 2014 | [14] | y | y | y | y | y | n | y | py | y | n | | 2 | 7 | 1 | 0 | Yes |
| Moreno-Conde et al., 2015 | [15] | y | y | y | y | y | py | y | y | y | y | | 0 | 9 | 0 | 1 | Yes |
| O'Donoghue et al., 2019 | [16] | y | y | y | n | n | n | y | y | y | y | | 3 | 7 | 0 | 0 | Yes |
| Shivade et al., 2014 | [17] | y | y | y | y | y | n | y | n | y | y | | 2 | 8 | 0 | 0 | Yes |
| Vazirani et al., 2019 | [18] | y | y | y | n | n | n | y | y | y | y | | 3 | 7 | 0 | 0 | Yes |
| Walsh et al., 2013 | [19] | y | y | y | y | y | n | y | y | y | y | | 1 | 9 | 0 | 0 | Yes |
| Wang et al., 2020 | [20] | y | y | y | y | y | n | y | n | y | y | | 2 | 8 | 0 | 0 | Yes |
| West et al., 2015 | [21] | y | y | y | n | n | n | y | n | y | y | | 4 | 6 | 0 | 0 | Yes |
| Xiao et al., 2018 | [22] | y | y | y | y | y | n | y | n | y | y | | 2 | 8 | 0 | 0 | Yes |
| Xu et al., 2015 | [23] | y | y | y | y | y | n | y | py | y | y | | 1 | 8 | 1 | 0 | Yes |
| Total number of Yes |  | 23 | 23 | 20 | 15 | 14 | - | 23 | 8 | 23 | 20 | | - | - | - | - | 20 |
| Total number of No |  | - | - | - | 5 | 5 | 21 | - | 12 | - | 3 | | - | - | - | - | 3 |
| Total number of partial yes |  | - | - | 3 | - | - | 2 | - | 3 | - | - | | - | - | - | - | - |
| Total number of unknown |  | - | - | - | 3 | 4 | - | - |  | - | - | | - | - | - | - | - |

n, no; y, yes; py, partial yes; unk, unknown.

**References**

1. Blackley SV, Huynh J, Wang L, Korach Z, Zhou L. Speech recognition for clinical documentation from 1990 to 2018: a systematic review. J Am Med Inform Assoc. 2019;26: 324–338.

2. Chukwu E, Garg L. A Systematic Review of Blockchain in Healthcare: Frameworks, Prototypes, and Implementations. IEEE Access. 2020;8: 21196–21214.

3. Dainton C, Chu CH. A review of electronic medical record keeping on mobile medical service trips in austere settings. Int J Med Inform. 2017;98: 33–40.

4. Dubovitskaya A, Novotny P, Xu Z, Wang F. Applications of Blockchain Technology for Data-Sharing in Oncology: Results from a Systematic Literature Review. Oncology. 2020;98: 403–411.

5. Hasselgren A, Kralevska K, Gligoroski D, Pedersen SA, Faxvaag A. Blockchain in healthcare and health sciences-A scoping review. Int J Med Inform. 2020;134: 104040.

6. Hussien HM, Yasin SM, Udzir SNI, Zaidan AA, Zaidan BB. A Systematic Review for Enabling of Develop a Blockchain Technology in Healthcare Application: Taxonomy, Substantially Analysis, Motivations, Challenges, Recommendations and Future Direction. J Med Syst. 2019;43: 320.

7. Juhn Y, Liu H. Artificial intelligence approaches using natural language processing to advance EHR-based clinical research. J Allergy Clin Immunol. 2020;145: 463–469.

8. Koleck TA, Dreisbach C, Bourne PE, Bakken S. Natural language processing of symptoms documented in free-text narratives of electronic health records: a systematic review. J Am Med Inform Assoc. 2019;26: 364–379.

9. Kreimeyer K, Foster M, Pandey A, Arya N, Halford G, Jones SF, et al. Natural language processing systems for capturing and standardizing unstructured clinical information: A systematic review. J Biomed Inform. 2017;73: 14–29.

10. Kumah-Crystal YA, Pirtle CJ, Whyte HM, Goode ES, Anders SH, Lehmann CU. Electronic Health Record Interactions through Voice: A Review. Appl Clin Inform. 2018;9: 541–552.

11. Mayer AH, da Costa CA, Righi R da R. Electronic health records in a Blockchain: A systematic review. Health Informatics J. 2020;26: 1273–1288.

12. Mazlan AA, Mohd Daud S, Mohd Sam S, Abas H, Abdul Rasid SZ, Yusof MF. Scalability Challenges in Healthcare Blockchain System—A Systematic Review. IEEE Access. 2020;8: 23663–23673.

13. Meystre SM, Friedlin FJ, South BR, Shen S, Samore MH. Automatic de-identification of textual documents in the electronic health record: a review of recent research. BMC Med Res Methodol. 2010;10: 70.

14. Mishra R, Bian J, Fiszman M, Weir CR, Jonnalagadda S, Mostafa J, et al. Text summarization in the biomedical domain: a systematic review of recent research. J Biomed Inform. 2014;52: 457–467.

15. Moreno-Conde A, Moner D, Cruz WD da, Santos MR, Maldonado JA, Robles M, et al. Clinical information modeling processes for semantic interoperability of electronic health records: systematic review and inductive analysis. J Am Med Inform Assoc. 2015;22: 925–934.

16. O’Donoghue O, Vazirani AA, Brindley D, Meinert E. Design Choices and Trade-Offs in Health Care Blockchain Implementations: Systematic Review. J Med Internet Res. 2019;21: e12426.

17. Shivade C, Raghavan P, Fosler-Lussier E, Embi PJ, Elhadad N, Johnson SB, et al. A review of approaches to identifying patient phenotype cohorts using electronic health records. J Am Med Inform Assoc. 2014;21: 221–230.

18. Vazirani AA, O’Donoghue O, Brindley D, Meinert E. Implementing Blockchains for Efficient Health Care: Systematic Review. J Med Internet Res. 2019;21: e12439.

19. Walsh C, Siegler EL, Cheston E, O’Donnell H, Collins S, Stein D, et al. Provider-to-provider electronic communication in the era of meaningful use: a review of the evidence. J Hosp Med. 2013;8: 589–597.

20. Wang Y, Wang L, Rastegar-Mojarad M, Moon S, Shen F, Afzal N, et al. Clinical information extraction applications: A literature review. J Biomed Inform. 2018;77: 34–49.

21. West VL, Borland D, Hammond WE. Innovative information visualization of electronic health record data: a systematic review. J Am Med Inform Assoc. 2015;22: 330–339.

22. Xiao C, Choi E, Sun J. Opportunities and challenges in developing deep learning models using electronic health records data: a systematic review. J Am Med Inform Assoc. 2018;25: 1419–1428.

23. Xu J, Rasmussen LV, Shaw PL, Jiang G, Kiefer RC, Mo H, et al. Review and evaluation of electronic health records-driven phenotype algorithm authoring tools for clinical and translational research. J Am Med Inform Assoc. 2015;22: 1251–1260.
