# Supplementary material for: Technological progress in electronic health record system optimization: Systematic review of systematic literature reviews
Source: Int J Med Inform. 2021 Aug;152:104507. doi: 10.1016/j.ijmedinf.2021.104507 (PMC8223493; doi:10.1016/j.ijmedinf.2021.104507)
Supplement: Supplementary file 5 [file mmc5.docx]

**Appendix D. Descriptive summary of the 23 systematic reviews included.**

| **Ref.** | **Author, year** | **Journal** | **Area** | **No. of studies included** | **Purpose** | **Relevant outcomes** |
| --- | --- | --- | --- | --- | --- | --- |
| [1] | Dubovitskaya et al., 2020 | Oncology | Blockchain Technology | 12 | This review analyzes the advantages, limitations, and challenges of blockchain technology for sharing EHRs data across providers, focusing on oncology. | Blockchain technology has the potential to enhance data-sharing thanks to transparency, traceability, and immutability. Blockchain needs to be combined with cryptographic techniques to guarantee data privacy and security. Evaluations in real-world settings and further research on privacy-preserving hybrid data storage, interoperable infrastructures, and architecture are still needed. |
| [2] | Hasselgren et al., 2020 | Int. J. Med. Inform. | Blockchain Technology | 39 | A systematic overview of studies that propose blockchain technology for optimizing healthcare processes. | Blockchain-based solutions are currently being explored in EHRs, Personal Health Records, and mobile health use cases, with Ethereum followed by Hyperledger as the most used platforms. A consortium blockchain is the preferred design choice. More research is needed in other domains of health information systems such as knowledge infrastructures, picture archiving, automated diagnostic service, administrative systems, or pharma supply chains. |
| [3] | Mayer et al., 2020 | Health Informatics J. | Blockchain Technology | 38 | Assessment of issues and potential benefits of Blockchain in healthcare. | Blockchain technology might improve EHR interoperability, establish sharing trust between healthcare providers, improve auditability, privacy, and granting health data access control by patients. However, the system's scalability is likely compromised because only users with large storage spaces and high computational power can partake in the Blockchain as full nodes. Solutions must be found to allow users to interact with the Blockchain, based on their available resources. |
| [4] | O'Donoghue et al., 2019 | J. Med. Internet Res. | Blockchain Technology | 15 | A systematic review of the trade-offs involved in different blockchain designs relevant to EHRs. | Many trade-offs involve improving blockchain at the expense of security, making data protection a critical factor for the system's effectiveness. Specific regulatory frameworks for effective blockchain-based EHRs must be developed. Multiple rule sets for exchanging data will allow for adaptive management and maximization of the utility of blockchain EHRs. Scalability could be improved, for instance, by moving data off-chain. Providers wishing to implement blockchain EHR systems should understand the current and future scale of their institution. |
| [5] | Vazirani et al., 2019 | J. Med. Internet Res. | Blockchain Technology | 71 | Summary of published evidence related to the implementation of blockchains to manage EHRs. | Data privacy remains one of the biggest challenges. Regulatory guidelines and use standards are still needed to manage consented access to EHRs and increase interoperability while protecting privacy. Private or consortium-led regulated blockchains could effectively address these issues. Stakeholders need to be educated on the benefits and the costs of transferring to the new system. |
| [6] | Hussien et al., 2019 | J. Med Syst. | Blockchain Technology | 58 | This study aimed to analyze and map the research landscape of  blockchain in healthcare applications. | Despite the significant potential of blockchain technology in healthcare, there are still security vulnerabilities and performance issues that need to be addressed. The impact of blockchain on EHRs remains a novel concept, and it is still in the documentation phase. Security, privacy, and scalability of blockchain need to be further developed. |
| [7] | Mazlan et al., 2020 | IEEE Access | Blockchain Technology | 38 | This review focused on the scalability challenges of blockchain in healthcare. | The number of nodes, transactions, the high volume of data, protocol challenges, and block size are important issues in implementing blockchain in healthcare. The review provides solutions for these issues, summarized in three for storage optimization and 13 for the redesign of the blockchain, namely blockchain modeling, read mechanism, write mechanism, and bi-directional network. |
| [8] | Chukwu et al., 2020 | IEEE Access | Blockchain Technology | 61 | The review assessed state-of-the-art blockchain applications in healthcare and compared them with traditional data management techniques. | Despite a surge of interest in blockchain applied to EHRs, research is still conceptual with minimal real-world application. Poor scalability, low performance, and high cost remain the most important challenges for effective implementation. Trust deficit might be a key factor responsible for the lack of progress in the implantation of blockchain technology in the healthcare sector. |

| **Ref.** | **Author, year** | **Journal** | **Area** | **No. of studies included** | **Purpose** | **Relevant outcomes** |
| --- | --- | --- | --- | --- | --- | --- |
| [9] | Mishra et al., 2014 | J. Biomed. Inform. | Information Extraction/ Natural Language Processing (NLP) | 34 | A systematic review of published research on the summarization of textual documents in the biomedical domain. | Natural Language Processing (17; 50%) and combined methods (15; 44%) were the most common summarization approaches. Research had shifted from a strong focus on single-document summarization to both single and multi-document summarization. Research was needed in the cognitive aspects of text summarization, including visualization techniques and evaluations of text summarization systems' impact in work settings. |
| [10] | Juhn et al., 2020 | J. Allergy Clin. Immunol. | Information Extraction/ Natural Language Processing (NLP) | 21 | Review of NLP techniques for extracting EHRs data for clinical research concerning allergy, asthma, and immunology. | NLP potentially enables automated chart review to reduce methodological heterogeneity in research. However, research work leveraging free texts in EHRs via NLP is severely limited. Challenges for NLP-based research to be addressed in the future are data quality issues, privacy issues, algorithmic bias, lack of interoperability standards, and HIS–related clinician burnout and workflow issues. |
| [11] | Koleck et al., 2019 | J. Am. Med. Inform. Assoc. | Information Extraction/ Natural Language Processing (NLP) | 27 | Summary of literature on the use of NLP to process symptom information documented in EHR free-text narratives. | Only half of the studies presented symptom information as a primary outcome, and 30% focused on using symptoms to classify disease. Open-source EHR-related NLP systems such as Apache cTAKESTM (ctakes.apache.org) or making expert-developed rule-based NLP algorithms openly available on platforms such as GitHub could facilitate transparency and replication of study findings, minimizing duplicated efforts. |
| [12] | Kreimeyer et al., 2017 | J. Biomed. Inform. | Information Extraction/ Natural Language Processing (NLP) | 86 | Compilation of a list of currently-in-use, complete NLP solutions for encoding free text into standardized clinical terminologies. | The review identified 71 different NLP systems that processed unstructured clinical text and generated structured output. Machine learning approaches are still rare but are growing in popularity. Nearly every system was focused on addressing a single clinical need. Real improvements in the field will come from making quality NLP applications available for specific use cases. |
| [13] | Wang et al., 2020 | J. Biomed. Inform. | Information Extraction/ Natural Language Processing (NLP) | 263 | Review of published research from 2009 to 2016 on clinical information extraction (IE) applications. | IE, considered a specialized area of NLP, has been underutilized for clinical and translational research. NLP had been dominated by rule-based approaches, which are nowadays regarded as obsolete compared to machine learning approaches. Efforts are needed to encourage NLP researchers to use EHR data. Additionally, access to EHR data should be improved, whereas semantics standardization needs to be adopted to enhance NLP systems' interoperability. |
| [14] | Kumah-Crystal et al., 2018 | Appl. Clin. Inform. | Speech recognition (SR) | 61 | This paper reviews the literature on voice input technology in EHR systems. | EHR interactions through voice are still evolving as an alternative to standard input methods. The emerging use of voice assistants in health care includes data retrieval, command execution, and chart navigation. SR can provide the opportunity to navigate EHR more productively, overcome constraints and inefficiencies imposed by classic graphical user interfaces and ultimately improve patient care. |
| [15] | Blackley et al., 2019 | J. Am. Med. Inform. Assoc. | Speech recognition (SR) | 122 | Review of the use of speech recognition technology for clinical documentation. | Speech Recognition (SR) technology is increasingly used for clinical documentation, mostly in emergency medicine and radiology, mainly through third-party vendors' SR systems. Existing SR research focuses on the impact of SR on documentation time/cost and productivity, the accuracy of SR, and the relationship between SR and traditional dictation and transcription. As SR-assisted documentation becomes prevalent, clinicians have expressed concerns about its accuracy, completeness, and potential impact on document quality. The results of the review are inconclusive regarding whether SR technology is helpful or a hindrance. Further research is needed to identify challenges and solutions for continuous improvement and understand its usability and impact on documentation quality, efficiency, and cost. |
| [16] | Shivade et al., 2014 | J. Am. Med. Inform. Assoc. | Phenotyping | 97 | Summary of literature describing approaches for automatically identifying patient cohorts with a specific phenotype. | Several different attempts at electronic phenotyping were identified, such as rule-based systems, NLP, semantic web technologies, and decision tree-based algorithms. Despite the growing trend in the areas of machine learning, rule-based systems are still dominant. The review found that there is still a lack of well-established solutions for identifying patient cohorts. |

| **Ref.** | **Author, year** | **Journal** | **Area** | **No. of studies included** | **Purpose** | **Relevant outcomes** |
| --- | --- | --- | --- | --- | --- | --- |
| [17] | Xu et al., 2015 | J. Am. Med. Inform. Assoc. | Phenotyping | 24 | Review of available software tools for authoring EHR-driven phenotype algorithms. | Phenotype authoring tools can provide interfaces that are relatively accessible for clinical researchers who may not have high expertise in database and query coding. However, many of the evaluated tools did not support complex logic specifications, unstructured data processing, and external analytic software. |
| [18] | Meystre et al., 2010 | BMC Med. Res. Methodol. | De-identification | 18 | Review of research in automatic de-identification of narrative text documents in EHRs. | The potential risks of re-identification increase along with the rise in patient data. Methods that performed well to de-identify text include machine learning approaches based on Conditional Random Fields, Decision Trees, Maximum Entropy models, or Support Vector Machines combined with dictionaries and sometimes regular expressions. However, de-identification might have some effect on NLP system performance. |
| [19] | West et al., 2015 | J. Am. Med. Inform. Assoc. | EHR visualization tools | 18 | The review investigated the use of visualization techniques reported between 1996 and 2013 and evaluated innovative approaches to information visualization of EHR data. | The review concluded that there are few innovative EHR visualization techniques that lend themselves to a large amount of data available electronically. The most common visualization techniques mentioned in the literature are LifeLines and KNAVE-II/VISITORS. Challenges include the following: the amount and complexity of EHR data; ability to use temporal data; uncertain data; missing values, quality, inaccurate data entry; mixed data types; ease of interpretation and intuitiveness; the interfaces and roles of the users; and evaluation of quality and effectiveness of the design and user satisfaction. |
| [20] | Dainton et al., 2017 | J. Med. Internet Res. | EMR systems for remote settings | 2 | Review of digital solutions for EHR systems specifically for its use by mobile medical teams in austere settings. | Digital solutions for EHR systems explicitly designed for austere settings are still in the early stages. 15 EHR systems were identified. Open MRS software had the greatest potential in this type of settings because of accessibility and wide adoption. The availability of numerous independent EMR systems may further fragment medical care in low resource settings. Further development is required considering as priority interoperability and data sharing with larger systems. |
| [21] | Moreno-Conde et al., 2015 | J. Am. Med. Inform. Assoc. | Clinical Information Models (CIMs) | 36 | This systematic review aimed to identify and compare processes and methodologies for defining CIMs, promoting EHRs interoperability (from 2000 to 2013). | CIMs are one of the essential aspects of the creation of standardized and interoperable EHR systems. A standard clinical information modeling process (CIMP) did not exist in the literature. The review showed that it is possible to create a standard methodology for developing CIMs, despite using different technologies and standards (e.g., EN ISO 13606 and openEHR, using archetypes, or HL7 v3, using templates) .Defining a unified good practice methodology to be used by any clinical information modeler is viable. |
| [22] | Walsh et al., 2013 | J. Med. Internet Res. | Provider-to provider electronic communication tools | 25 | The review assessed electronic communication tools (as supported within or external to an EHR) between healthcare providers (data exchange, communication, and care coordination). | Intra-EHR communication did not prove useful for care coordination. The following concerns regarding EHRs adoption were reported: (1) a false sense of security may reduce verbal communications; (2) fewer conversations within a practice can reduce both knowledge sharing and basic social interactions necessary for collaboration; and (3) privacy and confidentiality. |
| [23] | Xiao et al., 2018 | J. Am. Med. Inform. Assoc. | Deep learning | 98 | This review summarized all deep learning studies using EHR data from 2010 to 2018. | Compared to other machine learning approaches, deep learning models excel in modeling raw data and analytical tasks. However, several issues still exist, such as data and label quality and availability, interpretability, transparency, and ease of deployment. Furthermore, deep models often do not explicitly capture uncertainties. It is still difficult to access large EHR datasets to integrate deep EHR models into the current systems. |

**References**

1. Dubovitskaya A, Novotny P, Xu Z, Wang F. Applications of Blockchain Technology for Data-Sharing in Oncology: Results from a Systematic Literature Review. Oncology. 2020;98: 403–411.

2. Hasselgren A, Kralevska K, Gligoroski D, Pedersen SA, Faxvaag A. Blockchain in healthcare and health sciences-A scoping review. Int J Med Inform. 2020;134: 104040.

3. Mayer AH, da Costa CA, Righi R da R. Electronic health records in a Blockchain: A systematic review. Health Informatics J. 2020;26: 1273–1288.

4. O’Donoghue O, Vazirani AA, Brindley D, Meinert E. Design Choices and Trade-Offs in Health Care Blockchain Implementations: Systematic Review. J Med Internet Res. 2019;21: e12426.

5. Vazirani AA, O’Donoghue O, Brindley D, Meinert E. Implementing Blockchains for Efficient Health Care: Systematic Review. J Med Internet Res. 2019;21: e12439.

6. Hussien HM, Yasin SM, Udzir SNI, Zaidan AA, Zaidan BB. A Systematic Review for Enabling of Develop a Blockchain Technology in Healthcare Application: Taxonomy, Substantially Analysis, Motivations, Challenges, Recommendations and Future Direction. J Med Syst. 2019;43: 320.

7. Mazlan AA, Mohd Daud S, Mohd Sam S, Abas H, Abdul Rasid SZ, Yusof MF. Scalability Challenges in Healthcare Blockchain System—A Systematic Review. IEEE Access. 2020;8: 23663–23673.

8. Chukwu E, Garg L. A Systematic Review of Blockchain in Healthcare: Frameworks, Prototypes, and Implementations. IEEE Access. 2020;8: 21196–21214.

9. Mishra R, Bian J, Fiszman M, Weir CR, Jonnalagadda S, Mostafa J, et al. Text summarization in the biomedical domain: a systematic review of recent research. J Biomed Inform. 2014;52: 457–467.

10. Juhn Y, Liu H. Artificial intelligence approaches using natural language processing to advance EHR-based clinical research. J Allergy Clin Immunol. 2020;145: 463–469.

11. Koleck TA, Dreisbach C, Bourne PE, Bakken S. Natural language processing of symptoms documented in free-text narratives of electronic health records: a systematic review. J Am Med Inform Assoc. 2019;26: 364–379.

12. Kreimeyer K, Foster M, Pandey A, Arya N, Halford G, Jones SF, et al. Natural language processing systems for capturing and standardizing unstructured clinical information: A systematic review. J Biomed Inform. 2017;73: 14–29.

13. Wang Y, Wang L, Rastegar-Mojarad M, Moon S, Shen F, Afzal N, et al. Clinical information extraction applications: A literature review. J Biomed Inform. 2018;77: 34–49.

14. Kumah-Crystal YA, Pirtle CJ, Whyte HM, Goode ES, Anders SH, Lehmann CU. Electronic Health Record Interactions through Voice: A Review. Appl Clin Inform. 2018;9: 541–552.

15. Blackley SV, Huynh J, Wang L, Korach Z, Zhou L. Speech recognition for clinical documentation from 1990 to 2018: a systematic review. J Am Med Inform Assoc. 2019;26: 324–338.

16. Shivade C, Raghavan P, Fosler-Lussier E, Embi PJ, Elhadad N, Johnson SB, et al. A review of approaches to identifying patient phenotype cohorts using electronic health records. J Am Med Inform Assoc. 2014;21: 221–230.

17. Xu J, Rasmussen LV, Shaw PL, Jiang G, Kiefer RC, Mo H, et al. Review and evaluation of electronic health records-driven phenotype algorithm authoring tools for clinical and translational research. J Am Med Inform Assoc. 2015;22: 1251–1260.

18. Meystre SM, Friedlin FJ, South BR, Shen S, Samore MH. Automatic de-identification of textual documents in the electronic health record: a review of recent research. BMC Med Res Methodol. 2010;10: 70.

19. West VL, Borland D, Hammond WE. Innovative information visualization of electronic health record data: a systematic review. J Am Med Inform Assoc. 2015;22: 330–339.

20. Dainton C, Chu CH. A review of electronic medical record keeping on mobile medical service trips in austere settings. Int J Med Inform. 2017;98: 33–40.

21. Moreno-Conde A, Moner D, Cruz WD da, Santos MR, Maldonado JA, Robles M, et al. Clinical information modeling processes for semantic interoperability of electronic health records: systematic review and inductive analysis. J Am Med Inform Assoc. 2015;22: 925–934.

22. Walsh C, Siegler EL, Cheston E, O’Donnell H, Collins S, Stein D, et al. Provider-to-provider electronic communication in the era of meaningful use: a review of the evidence. J Hosp Med. 2013;8: 589–597.

23. Xiao C, Choi E, Sun J. Opportunities and challenges in developing deep learning models using electronic health records data: a systematic review. J Am Med Inform Assoc. 2018;25: 1419–1428.
